# RING E3 ligase TRIM8 is structurally selective for UBE2N-mediated ubiquitination

**DOI:** 10.1016/j.jbc.2026.113642

**Published:** 2026-10-14

**Authors:** Jane Dudley-Fraser, Diego Esposito, Katrin Rittinger

**Affiliations:** Molecular Structure of Cell Signalling Laboratory, The Francis Crick Institute, London, UK

**Keywords:** TRIM protein, RING, E3 ubiquitin ligase, ubiquitin, ubiquitination, X-ray crystallography, NMR

## Abstract

RING domains catalyze ubiquitination by stabilizing a closed conformation of E2-ubiquitin (Ub) conjugates, with TRIpartite Motif (TRIM) proteins representing the largest subfamily of RING-type E3 ligases. TRIM proteins are implicated in many physiological processes and diseases. For example, TRIM8 perturbation has been variously linked to neurodevelopmental defects and several cancer types, attributed to its ubiquitination of a myriad of putative substrates. However, the molecular mechanisms behind these modifications are not yet clear. Here we show that TRIM8 has preferential functionality with the E2 enzyme UBE2N to generate K63-linked poly-ubiquitin chains. By solving the crystal structure of TRIM8 RING bound to a UBE2N-Ub conjugate, we discover that its non-canonical asparagine “linchpin” residue belies this selectivity by failing to form key contacts required to stabilize the closed conformation of other E2 enzymes. These findings not only open the door to exploring the impact of TRIM8-mediated K63 chain formation in health and disease but also add to a growing appreciation of the specificities of RING domain catalytic activity.

The TRIM family of proteins is made up of 76 members, defined by their conserved TRIpartite Motif domain structure, made up of a RING domain, one or two B-box domains, and a coiled-coil domain (1). TRIM C-terminal domains (CTDs), meanwhile, vary and dictate their grouping into 12 subclasses (I-XI and unclassified). These CTDs are believed to facilitate diverse interactions, including the selection of ubiquitination substrates across many biological processes, such as infection, differentiation, and oncogenesis 2, 3, 4, 5, 6. The RING domain of TRIMs is generally assumed to confer ubiquitin E3 ligase activity, albeit with nuances in this paradigm now coming to the fore: from their preferential function with certain E2s to the identification of catalytically inactive “pseudoligase” RING domains 7, 8, 9. E2 preference is particularly significant for RING-mediated catalysis as they dictate the ubiquitin chain linkage generated (10). Capitalizing on the potential of TRIMs as either therapeutic targets or components of targeted protein degraders (*e.g*., PROTACs), however, relies on an in-depth understanding of their mechanistic and regulatory features.

RING dimerization is a crucial aspect of the TRIM E3 ligase catalytic mechanism, where, following the E1-mediated loading of ubiquitin onto an E2 enzyme to form an E2-Ub thioester, the E2 binds one RING domain whilst the conjugated ubiquitin makes contacts with both RINGs in the dimer. This is required to stabilize the E2-Ub into a “closed” catalytic conformation, primed for subsequent ubiquitin discharge onto substrate lysine residues (Fig. 1*A*) 11, 12, 13, 14, 15. TRIM RING dimerization is mediated by the assembly of a four-helix bundle with α helices N- and C-terminal to the zinc co-ordinating cross-brace “core” of each RING domain, with TRIMs lacking these helices unable to dimerize or catalyze ubiquitination 7, 16. Previous structural analyses have revealed key residues and interfaces in TRIM RINGs that mediate E2-Ub contacts, including a so-called “linchpin” residue that interacts with both the E2 and ubiquitin 15, 17. All currently available TRIM RING/E2-Ub structures harbor an arginine residue in this linchpin position but, strikingly, 30 of 66 (45%) of RING-containing TRIM proteins have an alternative linchpin residue. The structural mechanisms underpinning the ubiquitin E3 ligase activity of these TRIMs is unknown.

**Figure 1:**
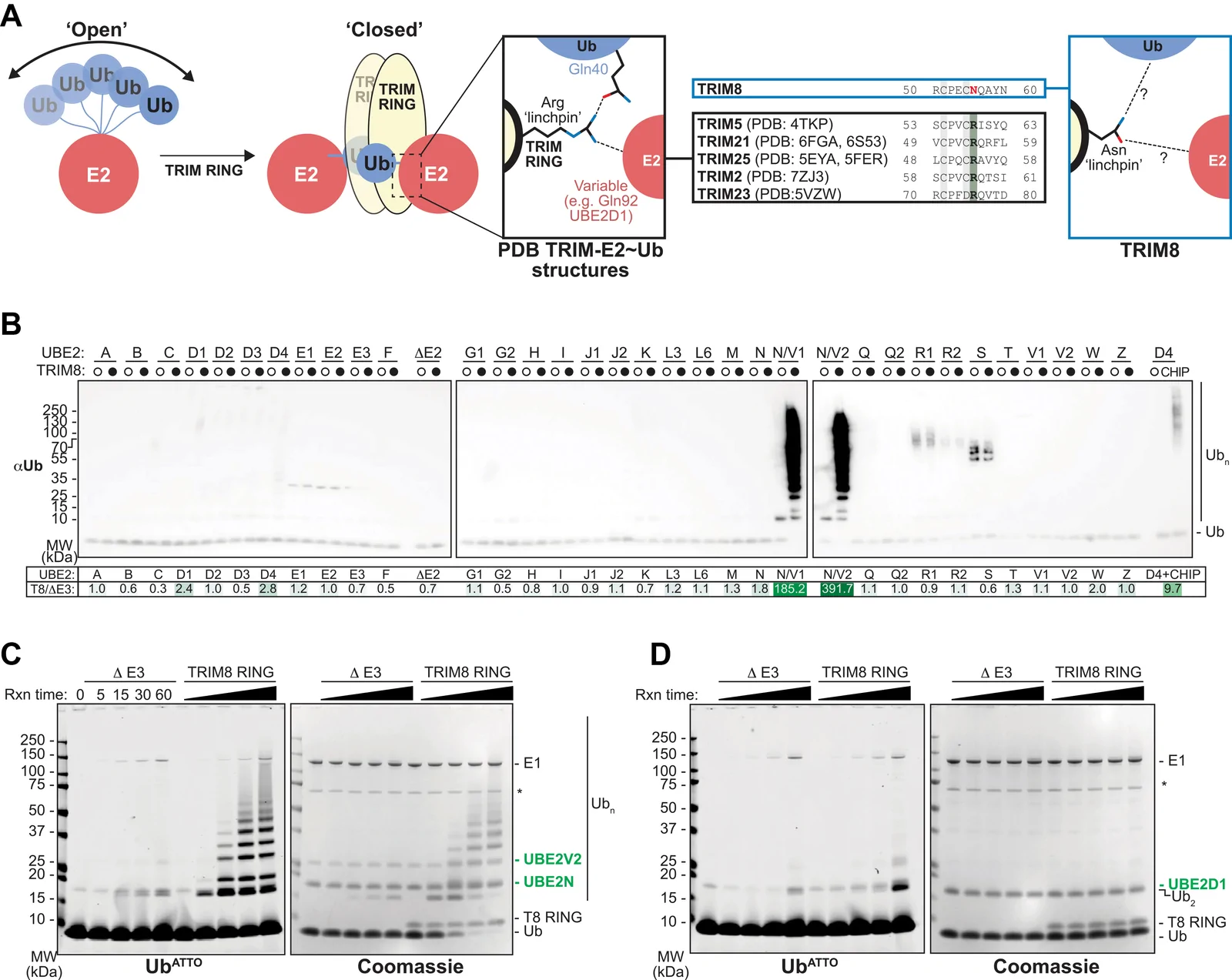
**TRIM8 exhibits strong ubiquitin E3 ligase activity with UBE2N but only marginal activity with UBE2D family members.***A*, schematic of stabilization of “closed”: E2-Ub conformation by TRIM RING dimers, as identified in previous TRIM/E2-Ub crystallographic structures in the PDB with arginine linchpin residues. *B*, Western blots against total ubiquitin of reaction products from E2^scan^ experiments in the presence (filled circles) or absence (unfilled circles) of TRIM8 RING (n = 1). Control experiments (ΔE2 or CHIP E3 ligase with UBE2D4) were used to validate the assay. Includes quantification of total ubiquitin signal (molecular weight > monomeric ubiquitin) for each lane, to reflect relative ubiquitination activity. *C-D*, fluorescent (Ub^ATTO^) and Coomassie gel scans of SDS-PAGE gels of ubiquitination reactions carried out for the specified length of time (min) at 30 °C using 2 μM TRIM8 RING, 50 μM ubiquitin, 1 μM UBA1, 1 μM Ub^ATTO^, and 3 mM ATP, alongside 2 μM UBE1N/V2 (C) or UBE2D1 (D) (n = 3).

TRIM8 is one such protein, with an asparagine residue at the linchpin position. TRIM8 is a class V TRIM, composed of a RING–B-box1–B-box2–coiled-coil motif followed by a stretch of 200 amino acids that are predicted to lack secondary structure but harbor a nuclear localization sequence (NLS) that mediates its organization into nuclear bodies 18, 19. Functionally, TRIM8 has been implicated in healthy brain development, with *de novo* TRIM8 truncation mutants resulting in childhood encephalopathy, as well as being originally dubbed Glioblastoma Expressed RING-finger Protein (GERP) due to its link to brain cancer 20, 21, 22, 23. On a cellular level, TRIM8 has also been linked to innate immune signalling, cilia regulation, proliferation, and promotion of stemness factors 24, 25, 26. These functions have been attributed to its ubiquitination of several substrates, including TAK1, MYOF, HNF1α, the PIAS3/STAT3 axis, as well as fellow family member TRIM21, with varying reports of TRIM8 catalysing formation of either K48- or K63-linked ubiquitin chains 25, 27, 28, 29, 30, 31. Thus far, however, mechanistically, it is unclear how substrate selection or ubiquitin linkage specificity are dictated. This is also the case more widely across the ubiquitin field, with cell-based studies frequently suggesting that RING E3s can generate specific ubiquitin linkages, likely by selective interactions with E2s that determine ubiquitin linkage type, but relatively few structures are available to explain the molecular mechanisms underpinning this.

Herein we demonstrate that the RING domain of TRIM8 preferentially functions with UBE2N to generate K63-linked chains. The crystal structure of TRIM8/UBE2N-Ub reported here indicates that this is due to the inability of the asparagine linchpin residue of TRIM8 to contact the E2. Consequently, whilst TRIM8 can catalyze ubiquitination with the closed conformation-favoring UBE2N (32), it is unable to stabilize the closed conformation of other E2s, including the promiscuous UBE2D family members (33). Evolutionarily, this appears to be a conserved feature of TRIM8 throughout metazoans, alluding to the importance of TRIM8-mediated K63-linked ubiquitination. Notably, these data highlight the idiosyncrasies of RING domains beyond the paradigm of non-specific E2-Ub scaffolding, and suggest a possible wider selectivity conferred by non-arginine linchpin TRIM RINGs.

## Results

### TRIM8 ubiquitin E3 ligase activity is selective for UBE2N to generate K63-linked ubiquitin chains

To investigate the E3 ligase activity of TRIM8, it was necessary to first establish which E2 enzymes it can function with. By carrying out an “E2^scan^” analysis (Ubiquigent, Dundee) containing all ubiquitin E2 conjugating enzymes and Western blotting for ubiquitin, recombinantly purified TRIM8 RING domain demonstrated a striking preference to generate ubiquitin chains with UBE2N/V1 and UBE2N/V2 heterodimeric E2 pairs (Fig. 1*B*). These results were confirmed in follow-up *in vitro* ubiquitination assays using fluorescently tagged ubiquitin (Ub^ATTO^, Figs. 1, *C*, *D* and S1, *A*–*F*). Interestingly, some weak reactivity was also observed with the highly promiscuous UBE2D family members (Figs. 1*D* and S1, *A*, *B*) (33). To explore the extent of its ability to function with UBE2D E2s, TRIM8 RING was tested in ubiquitination reactions with UBE2D1 (Fig. S1*G*) and assays measuring the RING-mediated discharge of CY5-labeled ubiquitin from UBE2D1 (Fig. S1, *H*–*J*). TRIM21 was used as a positive control as it functions with both E2s *in vitro* (9). These experiments demonstrate that in the presence of excess substrate, some TRIM8 ubiquitin E3 ligase activity with UBE2D1 can be detected but is negligible in comparison to its activity with UBE2N/V2 and relative to positive control TRIM21. Therefore, TRIM8 RING has a significant preference to function with UBE2N/V1 and UBE2N/V2, a heterodimeric E2 which synthesizes K63-linked polyubiquitin chains (34) (Fig. S2*A*).

### TRIM8 RING favors a monomeric state in solution

To understand the molecular basis for the selectivity of TRIM8, the crystal structure of TRIM8 RING in complex with UBE2N conjugated to ubiquitin (UBE2N-Ub, Fig. S2, *B* and *C*) was solved (Fig. 2*A*). Four copies of each molecule are present in the asymmetric unit (AU), belonging to the space group P 1 21 1 and refined to 2.4 Å (Table S1). The overall arrangement is consistent with that of previously solved TRIM RING/E2-Ub complexes, including those of TRIMs 2, 21, 23, and 25 9, 35, 36, 37, with each TRIM8 RING in the homodimer flanked by a UBE2N-Ub conjugate. Dimerization is mediated by a hydrophobic interface between α1 and α3 helices comparable to that of other TRIM RINGs (Fig. 2*B*). The TRIM8 RING dimer differs slightly, however, in the asymmetrical arrangement of these helices, with the top of α1 bent towards the core of the dimer in one monomer, likely due to the presence of the large tryptophan side chains at the N-terminus (Fig. 2*A*). Size exclusion chromatography coupled to multi-angle laser light scattering (SEC-MALLS, Fig. 2*C*) shows a monomer:dimer equilibrium in solution, similarly to TRIM2 RING, although for this latter it has been shown that the equilibrium is shifted towards the dimeric form (35). This existence of a monomer:dimer equilibrium was further supported by 2D NMR: the ^1^H-^15^N-HSQC spectrum of WT TRIM8 RING at 1 mM shows only a subset (∼40 cross peaks) of the construct total residues (74 amino acids, excluding proline residues), likely the result of line broadening due to chemical exchange between the RING monomeric and dimeric forms at an intermediate time scale (Fig. 2*D*). Interestingly, searching the PDB for structurally similar proteins using the tertiary amino acid interaction alignment tool FoldSeek (38) identifies the monomer-favoring TRIM25 RING structure (PDB 5FER (36), 5EYA (39)) as the most similar to TRIM8 (Fig. S3*A*).

**Figure 2:**
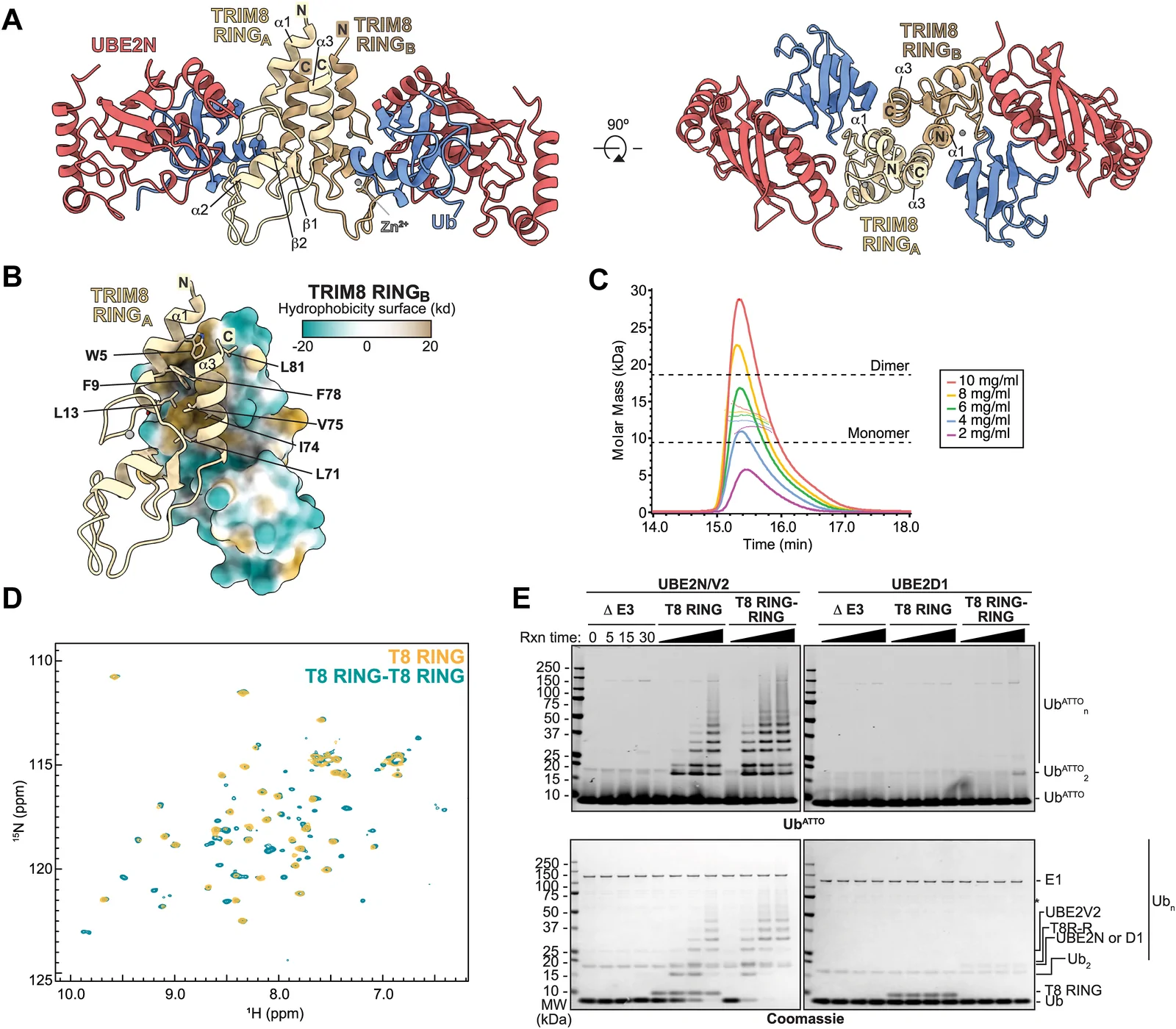
**TRIM8/UBE2N-Ub crystal structure and investigation of the dimerization properties of TRIM8 RING.***A*, cartoon representation of the crystal structure of the TRIM8 RING homodimer bound to two UBE2N-Ub conjugates. The *right panel* is related to the *upper* one by a 90° rotation about the *x*-axis. The two RING domains are represented in *light* and *dark beige*, whilst the E2 is in salmon and the ubiquitin in *blue*. The RING-co-ordinated Zn^2+^ atoms are represented as *grey spheres*. *B*, representation of the crystal structure of the TRIM8 RING homodimer as observed in the crystal structure of part A, with one RING shown as cartoon (*beige*) and the other a surface representation (coloured according to *low* (*teal*) to *high* (*yellow*) residue hydrophobicity (kd)). Residues from α1 and α3 helices that mediate RING dimerization are labeled. *C*, SEC-MALLS analysis of TRIM8 RING domains analysed at multiple concentrations, with molecular weights of monomeric and dimeric species indicated as dashed lines. *D*, natural abundance ^1^H-^15^N 2D HSQC spectrum of TRIM8 RING domain (*yellow*) or TRIM8 RING-RING tandem construct (*teal*). *E*, fluorescent (Ub^ATTO^) and Coomassie gel scans of SDS-PAGE gels of ubiquitination reactions carried out at 30 °C using 2 μM UBE2D1 or UBE2N/V2, 50 μM ubiquitin, 1 μM UBA1, 1 μM Ub^ATTO^, and 3 mM ATP, alongside 2 μM TRIM8 RING or 1 μM TRIM8 RING-RING tandem construct (n = 3).

Dimerization has previously been introduced synthetically using tandem RING-RING constructs 36, 40. Accordingly, a TRIM8 tandem construct where two RING domains are separated by a single serine residue shows a 2D ^1^H-^15^N-HSQC NMR spectrum that overlaps with the WT construct but contains a larger set of cross peaks, representing the spectrum of a single stabilized dimeric form. To assess whether the E2 preference of TRIM8 RING is related to its oligomerization state, the TRIM8 RING-RING tandem construct was used in *in vitro* ubiquitination assays with UBE2N/V2 or UBE2D1. These assays demonstrated that the tandem construct accelerated ubiquitin chain formation with UBE2N/V2 and increased di-ubiquitin production with UBE2D1, likely as a result of stabilizing RING dimer contacts that support a closed conformation of the E2-Ub thioester. The TRIM8 RING-RING tandem did not, however, significantly boost ubiquitin E3 ligase activity with UBE2D1 comparable to positive controls such as TRIM21 (Figs. 2*E* and S1*G*). These data demonstrate that TRIM8 RING exists in a monomer:dimer equilibrium and that TRIM8 E2 selectivity is not driven by oligomeric state.

### TRIM8 RING has extensive interactions with ubiquitin but limited E2 contacts

After ruling out the dimerization status of TRIM8 as the reason for its preferential function with UBE2N, the crystal structure of TRIM8 RING/UBE2N-Ub was further explored. Several interfaces between TRIM8 RING and ubiquitin were identified (Fig. 3*A*). TRIM8 residues E12, N73, and E76 in α1 and α3 helices form an “ionic string” of charge-based interactions with the side chains of K11 and K33 in ubiquitin; the conserved, zinc co-ordinating H32 of TRIM8 interacts with the backbone of G35 in ubiquitin; and the N56 alternative linchpin residue of TRIM8 contacts both the Q40 side chain and the backbone of R72 of ubiquitin. In other available TRIM/E2 structures, contacts between charged residues in these positions are also observed, including, but not exclusive to, components of a “tri-ionic anchor” identified in TRIM21/UBE2N-Ub by Kiss *et al.* (PDB 6S53 (9)). There also appears to be a degree of conservation throughout other TRIMs to exhibit charged or hydrogen bond-compatible residues at these positions (Fig. 3*C*). Conversely, in the only structure of a RING/E2-Ub in the PDB with an asparagine at the linchpin position, TRAF6/UBE2N-Ub, an asymmetric head-to-tail arrangement of the RING domains and an alternative interaction network are reported (PDB 5VNZ (41)). Although the ubiquitin residues involved are the same (K11, K33, Q40, R72), the residues in TRAF6 contributing to the interaction differ (G87, R126, E145, R147) (Fig. S4*A*).

**Figure 3:**
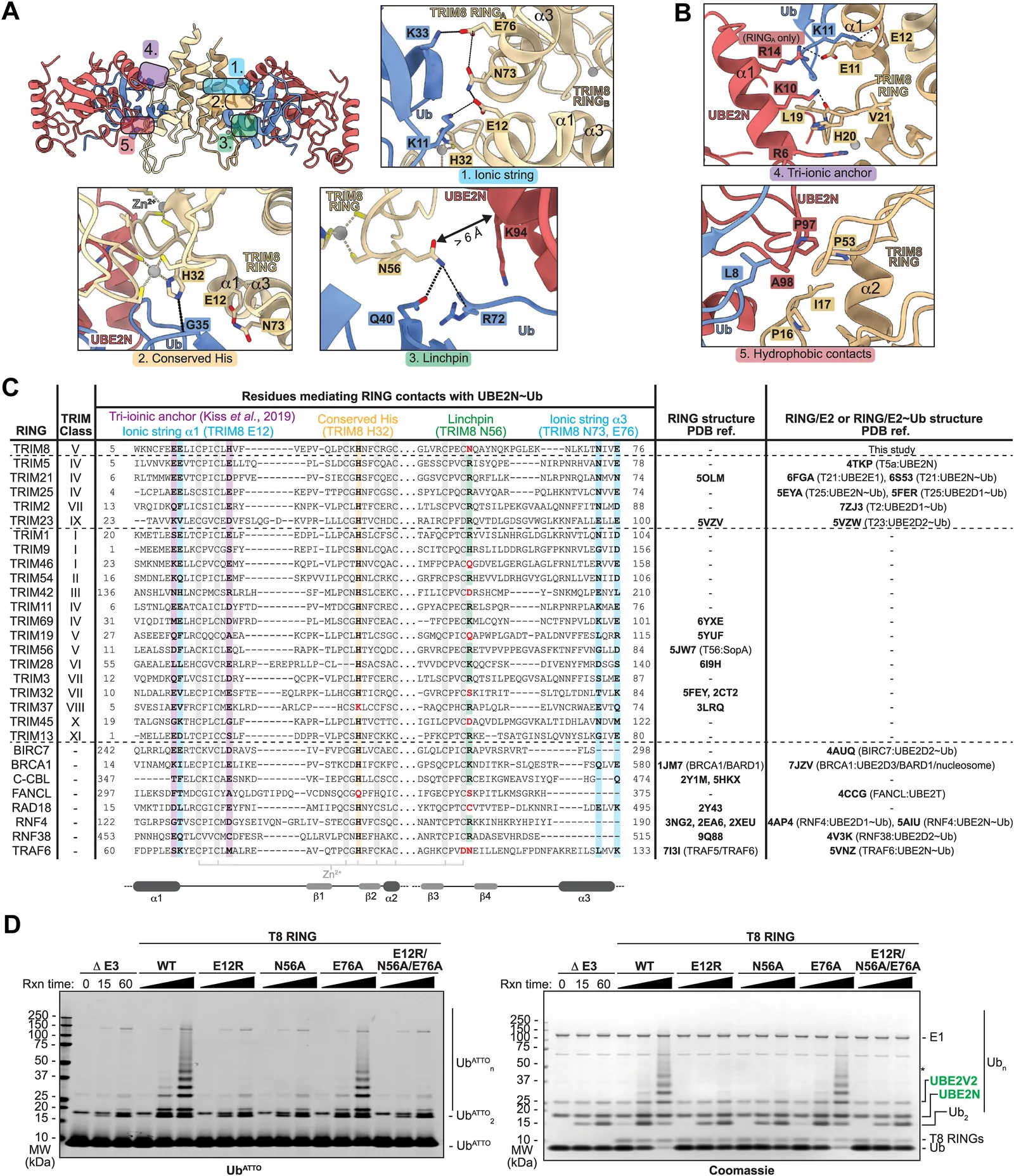
**Investigation of key interfaces in the TRIM8/UBE2N-Ub structure.***A–B*, cartoon representation of the crystal structure of the TRIM8 RING homodimer bound to two UBE2N-Ub conjugates with focussed views on key interfaces mediating RING-Ub (*A*) or RING-UBE2N (*B*) contacts. The two RING domains are represented in *light* and *dark beige*, whilst the E2 is in salmon and the ubiquitin in *blue*. The RING-co-ordinated Zn^2+^ atoms are represented as *grey spheres*. *C*, sequence alignments of RING domains of TRIMs across different classes and key examples RINGs with structures available in the PDB. Key features identified in parts (*A*-*B*) are indicated by colour coding (*blue* - ionic string; *yellow* - conserved His; *green* - linchpin; *purple* - tri-ionic anchor). *D*, fluorescent (Ub^ATTO^) and Coomassie gel scans of SDS-PAGE gels of ubiquitination reactions carried out at 30 °C using 2 μM UBE2N/V2, 50 μM ubiquitin, 1 μM UBA1, 1 μM Ub^ATTO^, and 3 mM ATP, alongside 2 μM wild-type (WT) or mutant TRIM8 RING domains (n = 3).

The TRIM8/UBE2N-Ub structure reveals relatively few contacts between TRIM8 RING and UBE2N (Fig. 3*B*). In both TRIM8 RING molecules, the L19 backbone carbonyl hydrogen bonds with the UBE2N K10 side chain and P16, I17, and P53 form a hydrophobic patch that makes contacts with UBE2N P97 and A98. The TRIM8 E11 side chain forms an ionic interaction with R14 in UBE2N in only one of the RING monomers (the “tri-ionic anchor” proposed by Kiss *et al.* (9)). Previous mutational analyses have demonstrated the importance of these TRIM RING/E2 contacts 9, 36. Unlike other TRIM RING/E2-Ub structures, however, the N56 alternative linchpin of TRIM8 is over 6 Å from the nearest atom of the E2. Interestingly, this is also what is observed in the structure of arginine linchpin-containing TRAF6 RING/UBE2N-Ub (Fig. S4*A*). Alignment of human E2 enzymes using a clustered Basic Local Alignment Search Tool (BLAST) search suggests that although the combination of the identity of these residues is unique to UBE2N, the closest paralogues are the UBE2D family, perhaps explaining why some low-level activity can be observed with these E2s (Fig. S4*B*). It is also notable that six of the top 20 PDB FoldSeek structural alignment hits of TRIM8 RING (excluding duplicate assembly entries) are of RING domains bound to UBE2N (Fig. S3*A*).

To probe the relative importance of the interactions between TRIM8 RING and ubiquitin, E12R, N56A, and E76A mutants of TRIM8 RING were generated. Whilst E12R and N56A almost completely inhibited ubiquitin chain formation, E76A only marginally reduced TRIM8 catalytic activity (Fig. 3*D*). A E12R/N56A/E76A triple mutant, meanwhile, completely ablated ubiquitination activity (Fig. S4*C*). Structural integrity of RING mutants was confirmed by circular dichroism (CD) spectroscopy (Fig. S4*D*). Therefore, these residues are required to mediate TRIM8 RING interactions with ubiquitin in order to catalyse ubiquitin discharge from UBE2N.

### Role of the “linchpin” residue in dictating TRIM RING:E2 interactions

Our crystal structure reveals that the asparagine residue at the linchpin position in TRIM8 (N56) only contacts the ubiquitin molecule in the E2-Ub complex and not the E2 enzyme, in contrast to all previously solved TRIM RING/E2-Ub structures where an arginine linchpin makes simultaneous contacts with ubiquitin and the E2 enzyme. We attribute this to the insufficient length of the residue, as TRIM8 N56R substitution in structural predictions of the complex with UBE2N-Ub or UBE2D1-Ub brings the residue in proximity with the backbone of the E2 (Q92 in UBE2N, K94 in UBE2D1) that interacts with the linchpin residue in available TRIM RING/E2-Ub structures (Fig. 4*A*). It is well established that the role of the RING arginine linchpin residues is to stabilize E2 closed conformations, with their mutation often ablating E3 ligase activity 9, 14, 17, 36, 42, 43. However, E2-Ub conjugates exist in open-closed conformational equilibria in solution, with UBE2N-Ub favoring the closed conformation even in the absence of an E3 ligase, whilst other E2s, such as UBE2D family members, exist predominantly in a more open conformation and require RING E3s to stabilize the closed confirmation 17, 32. Therefore, we hypothesized that TRIM8 selectively functions with UBE2N and fails to function effectively with UBE2D family members as it is unable to shift the open-closed conformation equilibrium sufficiently towards the closed state with its asparagine linchpin residue. To test this, a TRIM8 N56R RING domain mutant was employed in ubiquitin discharge and ubiquitination assays with UBE2D1. These data clearly show that TRIM8 N56R can function with UBE2D1, suggesting that it is more efficient at stabilizing the E2-Ub closed state (Figs. 4, *B*–*D* and S4, *E*–*G*). Interestingly, 2D ^1^H-^15^N HSQC spectra demonstrate that WT TRIM8 is able to interact with UBE2D3 to a similar extent to the N56R mutant, in agreement with the high degree of conservation of residues between UBE2D family members and UBE2N that mediate the interaction with TRIM8 (Figs. S4*B* and S5, *A*, *B*). However, to induce discharge of Ub from the E2-Ub conjugate, the interaction between RING and E2 must also stabilize the E2-Ub closed state. To investigate the conformational changes of ubiquitin in UBE2D1-Ub and UBE2N-Ub conjugates in the presence of TRIM8, 2D ^1^H-^15^N HSQC NMR spectra were recorded on samples of ^15^N-labeled Ub conjugated to either E2. In line with their ability to catalyze ubiquitination with UBE2N/V2, WT and N56R TRIM8 RING can both stabilize the UBE2N-Ub closed conformation, as evidenced by line broadening of the majority of the ^15^N-Ub resonances upon addition of equimolar amounts of RING (Figs. 4*E* and S5*C*). In contrast, addition of TRIM8 WT to UBE2D1-^15^N-Ub results in a lesser degree of spectral line broadening, suggesting reduced UBE2D1-Ub closed conformation stabilization, than TRIM8 N56R. Notably, the cross peak of residue I44, part of the hydrophobic patch of ubiquitin that contacts the E2 crossover helix in the closed state, is only slightly affected by the presence of TRIM8 WT, whereas it disappears by the addition of TRIM8 N56R 10, 32. These data suggest that TRIM8 works preferentially with UBE2N owing to the inability of its non-canonical asparagine linchpin to stabilize the closed conformation of other E2 enzymes, such as UBE2D isoforms. Interestingly, a clustered BLAST search of TRIM8 RING reveals it is highly conserved throughout metazoans, including the asparagine linchpin (Fig. S6, Table S2). Together, these findings demonstrate that TRIM8 has evolved from the TRIM family in metazoans to specialize in the generation of K63-linked ubiquitin chains using UBE2N.

**Figure 4:**
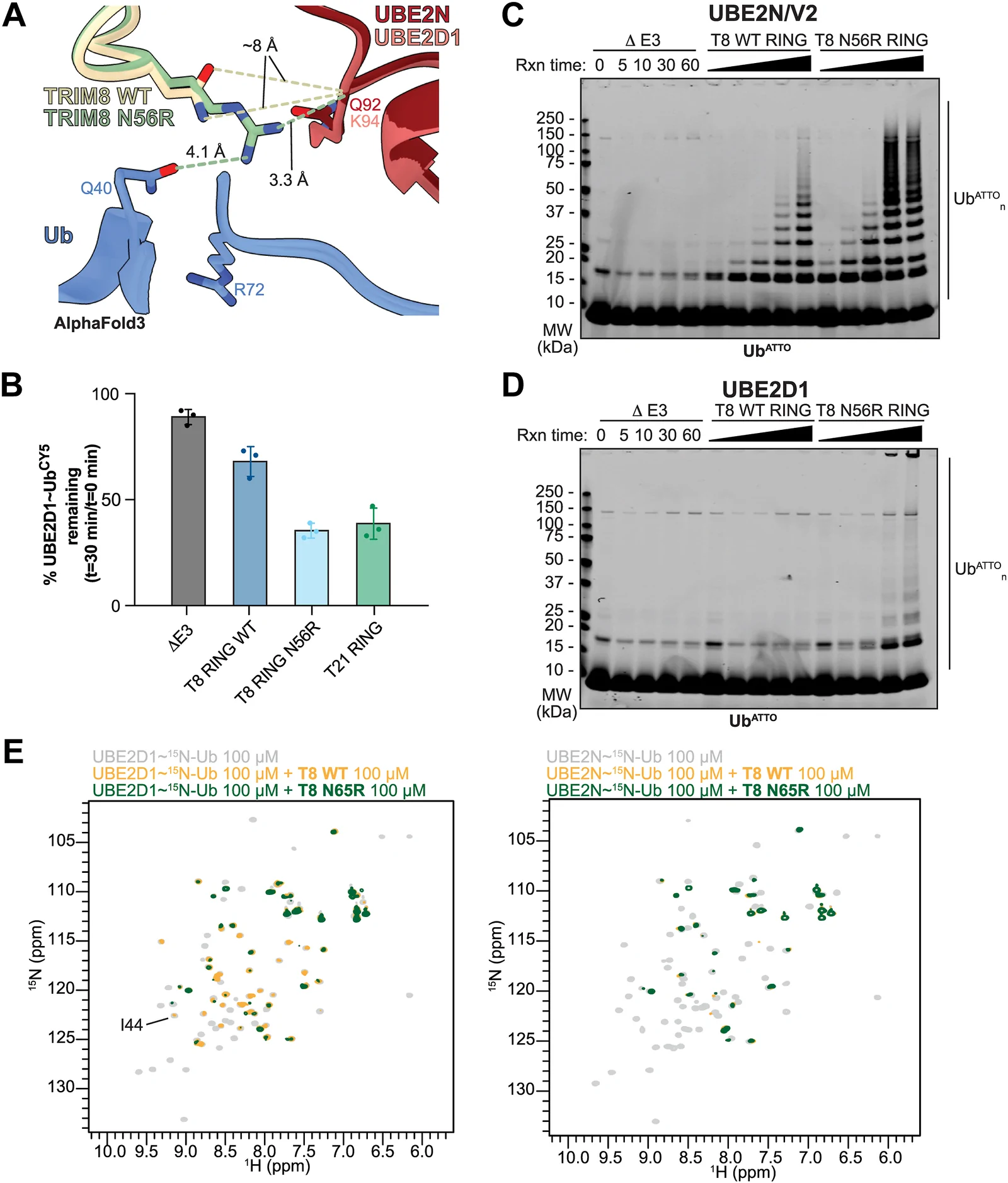
**Exploring the significance of the TRIM8 asparagine linchpin residue.***A*, AlphaFold3 structural predictions of RING domains of WT (*beige*) or N56R mutant (*green*) with ubiquitin (*blue*) and either UBE2N (*dark red*) or UBE2D1 (*salmon*) with key residues highlighted. Note, RINGs input as dimers with Zn^2+^. *B*, quantification of Ub^CY5^ discharge from pre-charged UBE2D1 onto 1 mM free lysine in solution over time at 25 °C, with 2 μM WT or N56R mutant TRIM8 RING or TRIM21 RING (see Fig. S4*G*, individual data points for n = 3 independent experiments represented by circles). *C-D*, fluorescent (Ub^ATTO^) gel scans of SDS-PAGE gels of ubiquitination reactions carried out at 30 °C using 2 μM UBE2N/V2 (*C*) or UBE2D1 (*D*), 50 μM ubiquitin, 1 μM UBA1, 1 μM Ub^ATTO^, and 3 mM ATP, alongside 2 μM wild-type (WT) or N56R mutant TRIM8 RING domains (n = 3, see also Fig. S4, *E* and *F*). *E*, ^1^H/^15^N spectra of ^15^N-labeled ubiquitin loaded onto UBE2D1-Ub or UBE2N-Ub without (*grey*) or with TRIM8 RING WT (*yellow*) or N56R mutant (*green*).

## Discussion

Deciphering the molecular mechanisms underlying the specificity of ubiquitin E3 ligases is crucial to understanding their physiological functions and the pathological consequences of their perturbation. In particular, it remains poorly understood how the majority of RING domains select E2s to generate specific types of ubiquitin chain linkage. In this study we set out to interrogate the catalytic properties of TRIM8 and uncovered a striking preference to generate K63-linked ubiquitin chains, thereby taking a first step towards understanding how its dysregulation can result in neurodevelopmental defects and brain cancer 20, 21, 22, 23.

Notably, we contribute to a growing body of work demonstrating that deviation from an arginine residue at the “linchpin” position of RING domains distorts ubiquitin E3 ligase activity (see Fig. 3*C* for alignments of the RINGs discussed below). For example, in a recent study, Huang and colleagues generated a comprehensive array of RNF38 linchpin point mutants and found that arginine was at least three-fold more effective in UBE2D2-mediated ubiquitination than any other residue (43). Similar to NMR data presented here, they observe that alternative linchpin residues induce insufficient UBE2D2-bound ^15^N-labeled ubiquitin NMR chemical shift perturbations to indicate a closed conformation. Interestingly, the crystal structure of FANCL:UBE2T (PDB 4CCG (44)) shows that its serine linchpin coordinates a water molecule to assist binding to the E2, in addition to other residues contributing to their cognate pairing. Similarly, whilst a structure for serine linchpin-containing TRIM32 RING dimer is available (PDB 5FEY (36)), it is unknown how it mediates contacts with ubiquitin in the E2-Ub conjugate.

Two examples of asparagine linchpin RING/E2-Ub structures are available, albeit demonstrating a significantly different arrangement to the TRIM8/UBE2N-Ub structure presented here. The first, TRAF6:UBE2N-Ub (PDB 5VNZ (41)) is explored above (see Fig. S4*A*). Of note, the RINGs are arranged in a head-to-tail dimer, in contrast to TRIM RING dimers. Secondly, a FoldSeek PDB structural homology search against TRIM8 RING identified RNF20 RING E3 ligase and its yeast orthologue Bre1 as highly similar, with both harboring an asparagine linchpin (RNF20 linchpin: 957-CPKCNAAFG-965) (Fig. S3*A*). Previous work in yeast has shown that Bre1 can modify polysomes after peroxide stress with K63-linked chains, which would represent an intriguing parallel with TRIM8/UBE2N (45). However, cryo-EM structures of RNF20 and Bre1 have been solved in complex with E2s which primarily function in histone mono-ubiquitination: UBE2A-Ub (PDB 8IEJ) and Rad6-Ub (PDB 8IEG), respectively (46). Notably, UBE2A/B do not exhibit any activity with TRIM8, and whilst TRIM8 N56A almost completely abolishes ubiquitin E3 ligase activity, the equivalent Bre1 N687A mutant does not (46). Unfortunately, the ubiquitin moiety is not visible in the RNF20-RNF40/UBE2A-Ub or Bre1/Rad6-Ub cryo-EM structures to discern how it contacts the RING domain.

Functionally, TRIM8-mediated K63-linked ubiquitination has been reported to modulate metabolic enzyme PGK1 and immune signaling protein TGFβ activated kinase 1 (TAK1, also known as MAP3K7) 25, 47. However, both of these targets reside outside the nucleus, which is at odds with previous work demonstrating a strong nuclear localization for TRIM8 as a result of a conserved nuclear localization sequence (NLS) in its C-terminal unstructured region (18). TRIM8 has alternatively been proposed to regulate two nuclear proteins: MDM2, by inducing its degradation to stabilize p53 (48); and PIAS3, by triggering its destabilization upon export from the nucleus and thus stimulating STAT3 signaling 29, 30. It remains to be clarified whether these are direct targets of TRIM8 ubiquitination and, if so, what chain types they are modified with.

Clinically, TRIM8 demonstrates frequent loss of heterozygosity in glioblastoma and the cProSite database (https://cprosite.ccr.cancer.gov/#/) reports that both TRIM8 and UBE2N are both downregulated in brain cancers 20, 49. Given the poor prognosis for glioblastoma, there is an urgent need to identify novel therapeutic approaches (50). Delineating the substrates of TRIM8/UBE2N K63-linked ubiquitination in these aggressive brain tumours may help characterize new drug targets. Further work is required to understand the normal physiological role of TRIM8, particularly in light of the intractable neurodevelopmental defects caused by mutations in its C-terminal region (22). The results presented here offer a valuable opportunity to begin to unravel TRIM8 functions through the understanding of its specialized ubiquitin E3 ligase activity.

## Experimental procedures

### Recombinant protein expression and purification

Cloning, expression, and purification of human E1 (UBA1) and E2 (UBE2D1 (WT or S22R/C85K), UBE2D2, UBE2D3 (WT or C85S), UB2E1, UBE2E2, UBE2K, UBE2N (WT or C87K/K92A), UBE2V2, UBE2R1) enzymes have been described previously 16, 35, 36, 51. UBE2D1-Ub^CY5^ was prepared as described previously 51, 52. Ubiquitin was purchased commercially (Sigma, U6253, from bovine erythrocytes). Human TRIM RING domains (TRIM21 R [1–85]; TRIM8 R [2–81]; TRIM8 R-R tandem [2–81]-serine-[2–81]) and their corresponding mutant constructs were generated as gBlocks (IDT) and ligated by Gibson Assembly into pET49b vector as HRV 3C protease cleavable N-terminal His_6_-fusion proteins (35). Briefly, the resulting His_6_-tagged proteins produced by BL21 (DE3) *E. coli* (grown in the presence of 200 μM ZnCl_2_ when generating RING domains) were purified by Ni-NTA affinity chromatography (ThermoFisher, 88222) in 50 mM HEPES pH 7.5, 300 mM NaCl, 20 mM imidazole, and 0.5 mM TCEP. This was followed by further purification by size-exclusion chromatography with Superdex S75 XK16/60 column (Cytiva) that had been pre-equilibrated with 50 mM HEPES pH 7.5, 150 mM NaCl, and 0.5 mM TCEP. All proteins were stored in a buffer of 50 mM HEPES pH 7.5, 150 mM NaCl and 0.5 mM TCEP.

### Generation of isopeptide-linked E2-Ub

The loading of ubiquitin (^15^N-labeled or unlabeled) onto either UBE2D1 S22R/C85K (for NMR analyses) or UBE2N C87K/K92A (for crystallographic and NMR analyses) *via* an isopeptide bond was performed to at least 70% E2-Ub/E2 conversion at 37 °C (∼4 h and ∼7 h, respectively), with 1 μM E1, 150 μM E2, and 200 μM ubiquitin in a buffer of 50 mM Tris pH 10.0, 150 mM NaCl, 5 mM MgCl_2_, 0.5 mM TCEP, and 3 mM ATP, as previously described 9, 36. Loaded E2-Ub species were then isolated by size exclusion chromatography, as specified earlier, in a buffer of 20 mM Tris pH 8.0, 150 mM NaCl.

### Ubiquitination assay

For *in vitro* ubiquitination assays, 2 μM recombinant TRIM E3 ligase constructs (or 1 μM in the case of TRIM8 RING-RING) purified from *E. coli* were combined with 1 μM E1, 2 μM of each indicated E2, 3 mM ATP, 50 μM ubiquitin (excepting, where indicated, saturation using 100 μM ubiquitin or spiking with 1 μM Ub^ATTO^), and reaction buffer (50 mM HEPES pH 7.5, 150 mM NaCl, 20 mM MgCl_2_). Reactions were incubated at 30 °C with agitation for the indicated times, then terminated by the addition of 4 x SDS sample buffer (Invitrogen) supplemented with 500 mM DTT and snap frozen. Samples were then analysed by detection of in-gel Ub^ATTO^ or CY5 fluorescence, where indicated, using a LI-COR CLx scanner, and Coomassie staining imaged using a BioRad ChemiDoc and analysis in ImageLab (v6.1.0). Quantification of TRIM8 RING-RING ubiquitination was plotted in GraphPad Prism (v11.1.0).

### Ubiquitin discharge assay

Lysine-discharge assays were carried out at 25 °C using 2 μM recombinant TRIM E3 ligase constructs purified from *E. coli* (as described above) incubated with 1 μM UBE2D1-Ub^CY5^ pre-charged thioester and 20 mM L-Lysine in reaction buffer (50 mM HEPES pH 7.5 and 150 mM NaCl). Samples were taken at stated time points by quenching with 2 x SDS sample buffer (Invitrogen) and snap-freezing, before resolving by SDS-PAGE and analysis on a LI-COR CLx scanner. Ubiquitin discharge (UBE2D1-Ub^CY5^/Ub^CY5^) was quantified by ImageLab Software (v6.1.0).

### Circular dichroism (CD) spectroscopy

Far-UV CD spectra were recorded between 260 and 190 nm on a JASCO J-815 CD spectrophotometer. TRIM8 RING WT and mutants were prepared in 15 mM phosphate buffer pH 7.5, 150 mM NaF, 0.5 mM TCEP, with a final protein concentration of 0.15 mg/ml. The spectra were acquired at 20 °C, with a scan speed of 20 nm/min, 25 accumulations and a path length of 1 mm. A spectrum for the buffer was recorded and subtracted from the protein spectra. Data were subsequently plotted using Prism (GraphPad, v10.2.3).

### SEC-MALLS

For size exclusion chromatography coupled to multi-angle laser light scattering (SEC-MALLS), 120 μl of 0.45 μm filtered TRIM8 RING domain sample at a range of concentrations (2–10 mg/ml to assess concentration-dependent self-association) was applied to Superdex 200 Increase 10/300 column attached to a Jasco HPLC system. The gel filtration columns were pre-equilibrated in 50 mM HEPES pH 7.5, 150 mM NaCl, 0.5 mM TCEP and 3 mM NaN_3_, and experimental runs were performed at a flow rate of 0.5 ml/min. Scattering intensity was measured by a DAWN-HELIOS II laser photometer (Wyatt Technology), while the refractive index was measured by an OPTILAB T-rEX differential refractometer (Wyatt Technology). The ASTRA software package (WYATT technology) was used to determine the average molecular mass and polydispersity across the individual elution peaks.

### Crystallization, data collection, phasing and refinement

TRIM8 RING and ubiquitin-conjugated UBE2N (C87K/K92A), both in 20 mM HEPES pH 7.5, 150 mM NaCl, 0.5 mM TCEP, were mixed at 200 μM concentration in a 1:1 ratio. Commercially available sitting drop crystallization screens were dispensed at 20 °C using an automated Mosquito machine (TTP Labtech). Crystals grew from a 100 nl protein solution plus 100 nl reservoir in 20% PEG3350, 0.2 M Ca acetate hydrate as precipitant. For X-ray data acquisition, crystals were cryoprotected with mother liquor containing 25% ethylene glycol. Diffraction data were collected on beamline IO4 (*λ* = 0.9537 Å) at the Diamond Light Source (Oxford, UK), processed using DIALS (53) and merged and scaled using AIMLESS (54). The structure of the complex was solved by molecular replacement using the AlphaFold3 prediction of the complex (55) as templates in Phenix Phaser (56). Models were iteratively improved by manual building in Coot (57) and refined using Refmac (58) and Phenix (56). Coordinates and structure factors are deposited in the Protein Data Bank under accession code 32WJ. Further details on data collection and refinement statistics are summarized in Table S1.

### NMR

Natural abundance ^1^H-^15^N HSQC NMR spectra of the TRIM8 RING (residues 2–81) or TRIM8 RING-RING tandem (duplicate of residues 2–81, linked by a serine residue) were acquired at 1 mM protein concentration in 20 mM HEPES pH 7.5, 50 mM NaCl and 0.5 mM TCEP at 298 K on a Bruker AVANCE spectrometer operating at a ^1^H frequency 800 MHz. The data were recorded using Topspin (Bruker) and processed with NMRPipe (59).

2D ^1^H-^15^N HSQC NMR experiments were carried out using ^15^N-isotope enriched WT ubiquitin (a.) or UBE2D3 C85S mutant (b.). Resonance assignment of ubiquitin was available in the laboratory. UBE2D3 C85S amide proton chemical shift assignment was kindly provided by Rachel Klevit. Labeled proteins were prepared by growing the bacteria in M9 minimal medium using 1 g/L of ^15^N-ammonium chloride as the sole source of nitrogen. Data were acquired with Topspin (Bruker), processed with NMRPipe (59) and analysed by CCPNMR (60).

a. ^15^N-labeled ubiquitin analysis was preceded by its conjugation *via* an oxyester bond to UBE2N (C87K/K92A mutant to prevent hydrolysis of the E2-Ub thioester or ubiquitin transfer onto K92 (61)), or UBE2D1 (S22R/C85K mutant to prevent thioester hydrolysis or “backside” ubiquitin binding on the E2 at S22 (62)). Spectra of labeled Ub loaded onto E2s were recorded similar to as previously described (16) at 298K at a constant concentration of labeled component and WT or N56R TRIM8 RING domains (residues 2–81) (100 μM:100 μM) on a Bruker AVANCE spectrometer operating at a proton frequency of 800 MHz in 25 mM Na-phosphate pH 7.0 and 150 mM NaCl. Spectra perturbations were mapped onto the available crystal structure of ubiquitin (PDB 1UBQ (63)).
b. Spectra of ^15^N-labeled UBE2D3 were recorded similarly as previously described (16) at 298K on a Bruker AVANCE spectrometer operating at a proton frequency of 800 MHz in 25 mM Na-phosphate pH 7.0 and 150 mM NaCl. Backbone amide proton and nitrogen nuclei chemical shifts perturbations (σ) were calculated using CCPNMR analysis (v2.4.2) (60) and mapped according to their value on the available crystal structure (PDB 5EGG (64)). All data were plotted using Prism (GraphPad, v10.2.3) and all molecular graphics were prepared using ChimeraX (v1.9).

### E2^scan^

An E2^scan^ (v2, Ubiquigent) assay of TRIM8 E2 preference was executed according to the manufacturer’s protocol with 0.3 μM E1, 2.5 μM E2s, 1 μM E3, 100 μM ubiquitin, and 2 mM ATP in 25 mM HEPES pH 7.5, 150 mM NaCl and 20 mM MgCl_2_ for 1 h at 30 °C. Samples were run alongside the PageRuler Plus molecular weight ladder (Thermo, 26619) on 4 to 12% gradient NuPAGE Bis-Tris gels (Invitrogen) in MES buffer, then transferred onto nitrocellulose membranes using the TransBlot Turbo system (BioRad). Membranes were blocked in 5% milk in PBS with 0.001% Tween 20 (PBST) before incubation with the specified antibodies prepared in 5% milk (anti-ubiquitin (Ubi-1: Invitrogen, 13–1600, 1:1000), which was detected by anti-mouse-HRP antibody (Cell Signaling Technologies, #7076, 1:2000). Finally, detection reagents (Amersham, RPN2106V1&2) were added before imaging with BioRad ChemiDoc and analysis in ImageLab, including quantification of total ubiquitin signal per lane (> molecular weight than monomeric ubiquitin) (v6.1.0).

### Sequence alignment, structural similarity, and structural prediction searches

Predictions of TRIM8 RING (WT and N56R, residues 1–81) interaction with ubiquitin alongside full-length WT UBE2D1 or WT UBE2N were generated using AlphaFold3(55).

To probe the evolutionary conservation of TRIM8 RING domain, its protein sequence (residues 1–81) was used as a search query on the FoldSeek (38) and NCBI ClusteredNR BLAST, with subsequent alignments analyzed in JalView (v2.11.5.0).

### Statistical analyses

Independent biological repeats of biochemical assays were conducted at least n = 3. Bar graphs represent the mean ± error bars of the standard error of the mean (SEM) where applicable.

## Data availability

Protein Data Bank accession codes used in this study: 5EGG (UBE2D3); 1UBQ (Ubiquitin); 5VNZ (TRAF6/UBE2N-Ub); 32WJ (TRIM8/UBE2N-Ub). All other data that support the conclusions in this study are available with the main Figures as well as Supplementary Figures and Tables.

## Supporting information

This article contains supporting information^41^, ^63^, ^64^.

## Conflict of interest

The authors declare that they have no conflicts of interest with the contents of this article.
