# Supplementary material for: RING E3 ligase TRIM8 is structurally selective for UBE2N-mediated ubiquitination
Source: J Biol Chem. 2026 Oct 14;302(12):113642. doi: 10.1016/j.jbc.2026.113642 (PMC13769585; doi:10.1016/j.jbc.2026.113642)
Supplement: Supplementary file 1 [file mmc1.pdf]

Supplementary Information for:

RING E3 ligase TRIM8 is structurally selective for UBE2N-mediated ubiquitination

Jane Dudley-Fraser[1], Diego Esposito[1], Katrin Rittinger[1]*

[1] Structure of Cell Signalling Laboratory, The Francis Crick Institute, 1 Midland Road, London, NW1 1AT, UK.

* Correspondence: katrin.rittinger@crick.ac.uk.

<u>Contents</u>

Figures S1 to S6	Pages S-1 to S-12
Tables S1 and S2	Pages S-13 to S-15

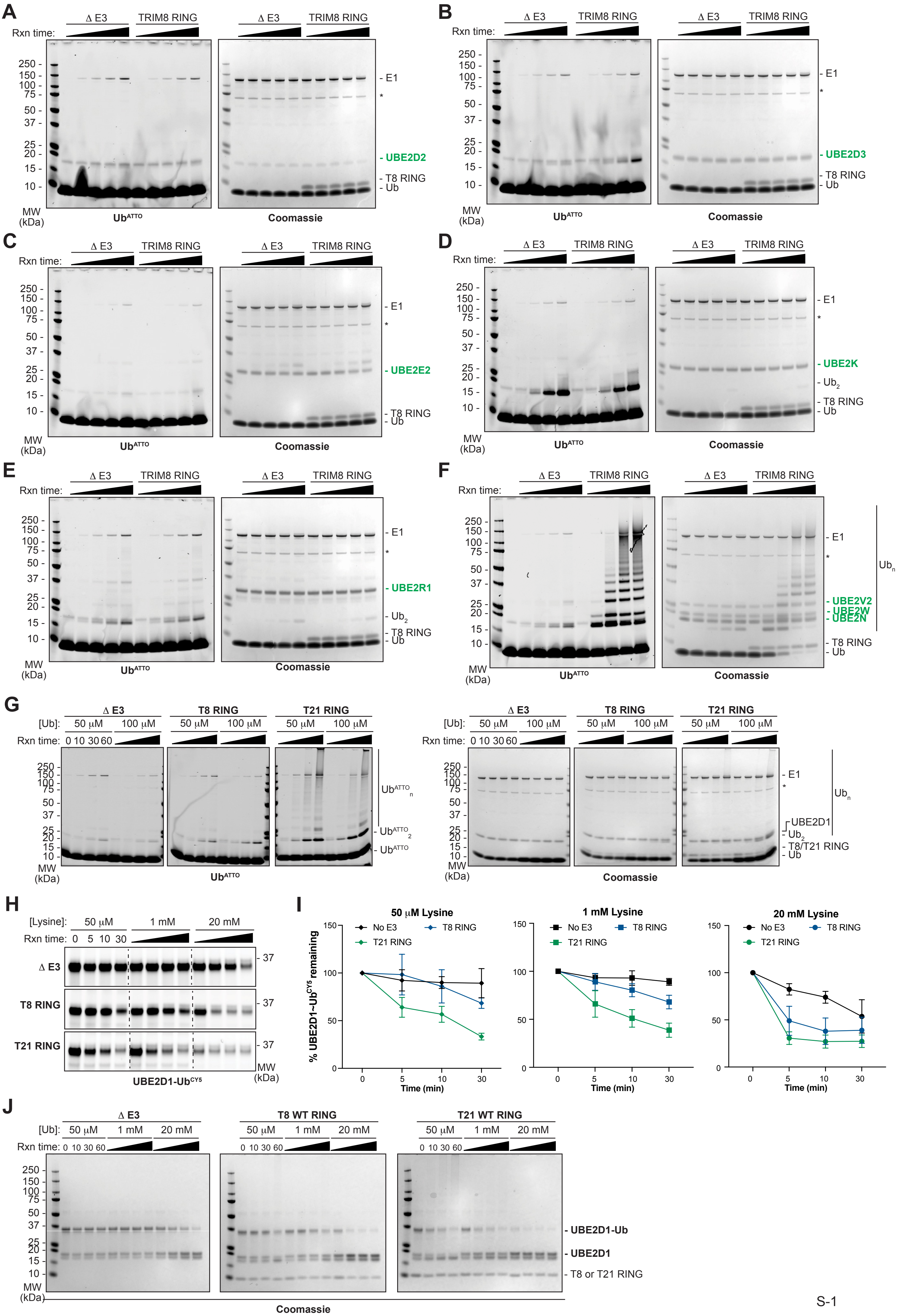
A
B
C
D
E
F
Δ E3
TRIM8 RING
Rxn time:
250 - 150 - 100 - 75 - 50 - 37 - 25 - 20 - 15 - 10 -
MW (kDa)
Ub^ATTO
Coomassie
- E1
*
- UBE2D2
- UBE2D3
- UBE2E2
- UBE2K
- UBE2R1
- UBE2V2
- UBE2W
- UBE2N
- Ub2
- T8 RING
- Ub
Ubn
G
[Ub]: 50 μM 100 μM
Rxn time: 0 10 30 60
T8 RING
T21 RING
Ub^ATTO n
Ub^ATTO 2
Ub^ATTO
UBE2D1
T8/T21 RING
H
[Lysine]: 50 μM 1 mM 20 mM
Rxn time: 0 5 10 30
Δ E3
T8 RING
T21 RING
- 37
UBE2D1-Ub^CY5
I
50 μM Lysine
1 mM Lysine
20 mM Lysine
No E3
T8 RING
T21 RING
% UBE2D1~Ub^CY5 remaining
150 100 50 0
0 5 10 30
Time (min)
J
T8 WT RING
T21 WT RING
[Ub]: 50 μM 1 mM 20 mM
Rxn time: 0 10 30 60
- UBE2D1-Ub
- UBE2D1
- T8 or T21 RING
Coomassie

Figure S1 – Follow-up validation of E2^{scan} results

Fluorescent (Ub^{ATTO}) and Coomassie gel scans of SDS-PAGE gels of ubiquitination reactions carried out for the specified length of time (min) at 30°C using 2 μM TRIM8 RING, 50 μM ubiquitin, 1 μM UBA1, 1 μM Ub^{ATTO} , and 3 mM ATP, alongside 2 μM different E2 enzymes (UBE2D2 (**A**), UBE2D3 (**B**), UBE2E2 (**C**), UBE2K (**D**), UBE2R1 (**E**), UBE2W/N/V2 (**F**) (n = 1). **G**, Fluorescent (Ub^{ATTO}) and Coomassie gel scans of SDS-PAGE gels of ubiquitination reactions carried out at 30°C using 2 μM UBE2D1, 1 μM UBA1, 1 μM Ub^{ATTO} , 3 mM ATP, and either 50 μM or 100 μM ubiquitin, alongside 2 μM TRIM8 or TRIM21 RING domains (n = 3). **H-J**, Fluorescent (Ub^{CY5}) gel scans (**H**), quantification of UBE2D1-Ub^{CY5} (**I**, plotted as mean values ± SEM, n=3), and Coomassie gel scans (**J**) of discharge assay of Ub^{CY5} from pre-charged UBE2D1 onto 50 μM, 1 mM, or 20 mM free lysine in solution over time at 25°C, with 2 μM TRIM8 or TRIM21 RING domains.

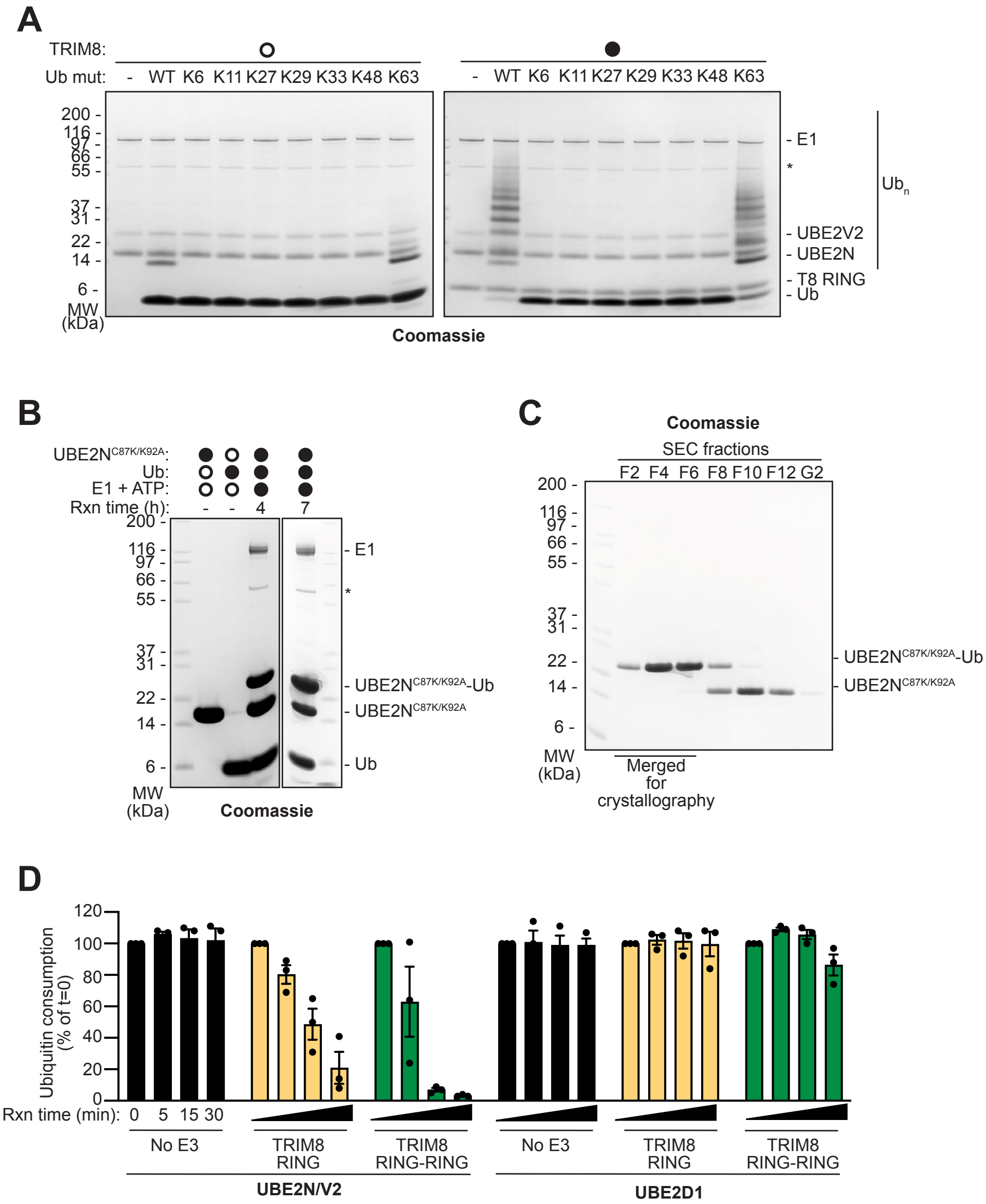

A
TRIM8:
Ub mut: - WT K6 K11 K27 K29 K33 K48 K63
200
116
97
66
55
37
31
22
14
6
MW (kDa)
E1
*
UBE2V2
UBE2N
T8 RING
Ub
Ubn
Coomassie
B
UBE2NC87K/K92A:
Ub:
E1 + ATP:
Rxn time (h): - - 4 7
E1
UBE2NC87K/K92A-Ub
UBE2NC87K/K92A
Ub
Coomassie
C
Coomassie
SEC fractions
F2 F4 F6 F8 F10 F12 G2
UBE2NC87K/K92A-Ub
UBE2NC87K/K92A
Merged for crystallography
D
Ubiquitin consumption (% of t=0)
Rxn time (min): 0 5 15 30
No E3
TRIM8 RING
TRIM8 RING-RING
UBE2N/V2
UBE2D1

Figure S2 – TRIM8 reacts with UBE2N/V2 to generate only K63-linked chains, preparation of UBE2N-Ub for crystallography

A, Coomassie gel scans of SDS-PAGE gels of ubiquitination reactions carried out for the specified length of time (min) at 30°C using 2 μM TRIM8 RING, 2 μM UBE2N/V2, 1 μM UBA1, and 3 mM ATP, alongside 50 μM WT or 'K only' mutant ubiquitin proteins where all lysine residues are mutated to arginine except for the one indicated (n = 4). **B**, Coomassie gel scans of SDS-PAGE gels of UBE2N C87K/K92A loading with ubiquitin. **C**, Size exclusion chromatography (SEC) fractions of ubiquitin-loaded UBE2N C87K/K92A, indicating the fractions combined and concentrated for use in crystallographic studies. **D**, Quantification of ubiquitin consumption (ubiquitin band volume/t=0 ubiquitin band volume (%)) with the TRIM8 RING or RING-RING constructs from Fig. 2E.

A Foldseek: TRIM8 RING (1-81) PDB tree

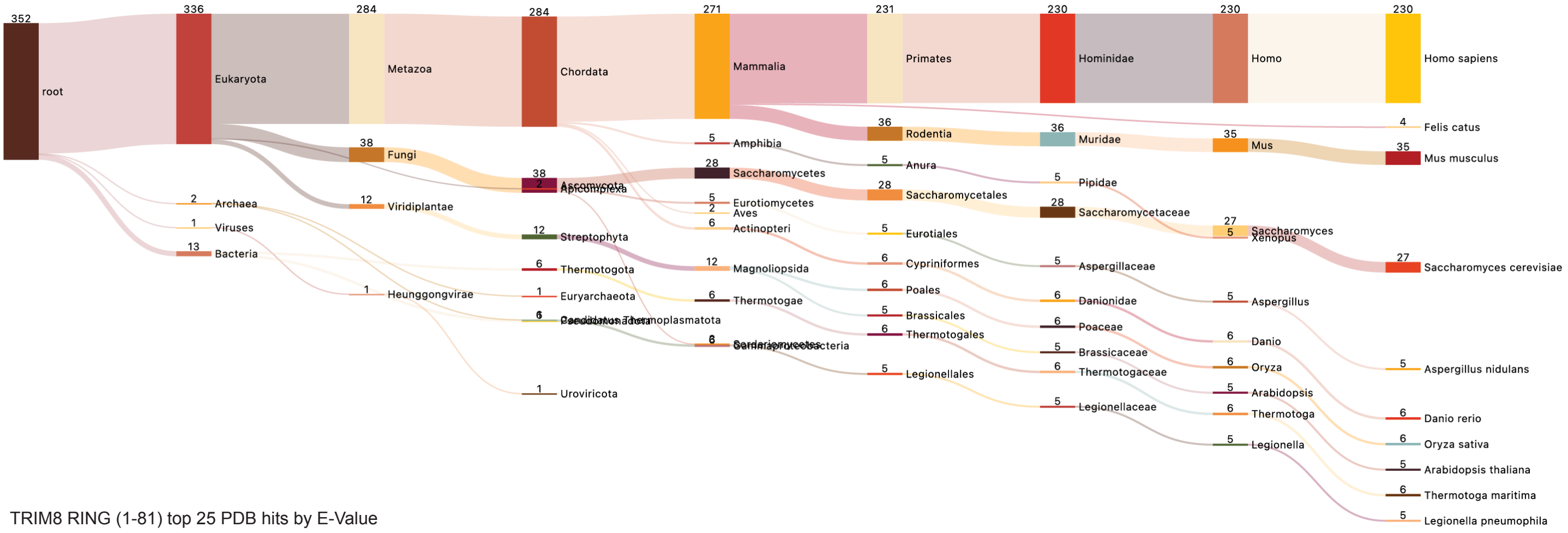

TRIM8 RING (1-81) top 25 PDB hits by E-Value

Target	Description	Scientific Name	Prob.	Seq. Id.	E-Value	Start	End
5fer-assembly1_A	Complex of TRIM25 RING with UbcH5-Ub	Homo sapiens	1	45.9	3.30E-08	8	81
5eya-assembly1_F	TRIM25 RING domain in complex with Ubc13-Ub conjugate	Homo sapiens	1	47.2	6.06E-08	9	80
5eya-assembly1_G	TRIM25 RING domain in complex with Ubc13-Ub conjugate	Homo sapiens	1	47.4	9.73E-08	1	78
6yxe-assembly2_B-2	Structure of the Trim69 RING domain	Homo sapiens	1	37.1	2.04E-06	8	77
8a58-assembly1_D	X-ray structure of TRIM21 RING E3 ligase in complex with E2 enzyme Ube2W	Homo sapiens	1	39.1	2.67E-06	7	80
8a58-assembly1_C	X-ray structure of TRIM21 RING E3 ligase in complex with E2 enzyme Ube2W	Homo sapiens	1	38.2	2.86E-06	1	80
6yxe-assembly1_A-3	Structure of the Trim69 RING domain	Homo sapiens	1	32.9	3.75E-06	1	81
8a38-assembly2_B	RING domain of human TRIM2	Homo sapiens	1	33.3	1.66E-05	13	77
6fga-assembly1_E	Crystal structure of TRIM21 E3 ligase, RING domain in complex with its cognate E2 conjugating enzyme UBE2E1	Homo sapiens	1	39.1	2.17E-05	7	80
7zj3-assembly2_A	Structure of TRIM2 RING domain in complex with UBE2D1~Ub conjugate	Homo sapiens	1	27.7	2.85E-05	4	81
6fga-assembly3_D	Crystal structure of TRIM21 E3 ligase, RING domain in complex with its cognate E2 conjugating enzyme UBE2E1	Homo sapiens	1	41.6	3.05E-05	10	81
6fga-assembly4_H	Crystal structure of TRIM21 E3 ligase, RING domain in complex with its cognate E2 conjugating enzyme UBE2E1	Homo sapiens	1	38.6	4.57E-05	7	80
8a38-assembly3_C	RING domain of human TRIM2	Homo sapiens	1	31.9	4.57E-05	10	77
4whv-assembly2_J	E3 ubiquitin-protein ligase RNF8 in complex with Ubiquitin-conjugating enzyme E2 N and Polyubiquitin-B	Homo sapiens	1	36.5	5.99E-05	2	81
6s53-assembly2_H	Crystal structure of TRIM21 RING domain in complex with an isopeptide-linked Ube2N~ubiquitin conjugate	Homo sapiens	1	38.6	6.41E-05	6	80
5olm-assembly1_A	TRIM21	Homo sapiens	1	38.3	7.85E-05	6	77
7bbd-assembly1_B	Crystal structure of monoubiquitinated TRIM21 RING with ubiquitin charged Ube2N (Ube2N~Ub) and Ube2V2	Homo sapiens	1	38.1	8.39E-05	5	80
7lyb-assembly1_M	Cryo-EM structure of the human nucleosome core particle in complex with BRCA1-BARD1-UbcH5c	Homo sapiens	1	30.3	8.98E-05	2	80
4orh-assembly1_L	Crystal structure of RNF8 bound to the UBC13/MMS2 heterodimer	Homo sapiens	1	35.8	8.98E-05	2	81
5olm-assembly1_B	TRIM21	Homo sapiens	1	39.7	1.18E-04	1	77
4orh-assembly3_C	Crystal structure of RNF8 bound to the UBC13/MMS2 heterodimer	Homo sapiens	1	37.1	1.18E-04	6	81
4ayc-assembly1_B-2	RNF8 RING domain structure	Homo sapiens	1	35.8	1.18E-04	2	81
8grq-assembly1_K	Cryo-EM structure of BRCA1/BARD1 bound to H2AK127-UbcH5c-Ub nucleosome	Homo sapiens	1	28.2	1.18E-04	2	80
4tkp-assembly1_B	Complex of Ubc13 with the RING domain of the TRIM5alpha retroviral restriction factor	Macaca mulatta	1	36.9	1.54E-04	6	78
4r8p-assembly1_K	Crystal structure of the Ring1B/Bmi1/UbcH5c PRC1 ubiquitylation module bound to the nucleosome core particle	Homo sapiens	1	25	1.54E-04	7	78

B Foldseek: TRIM8 RING (1-81) AlphaFold Database Proteome tree

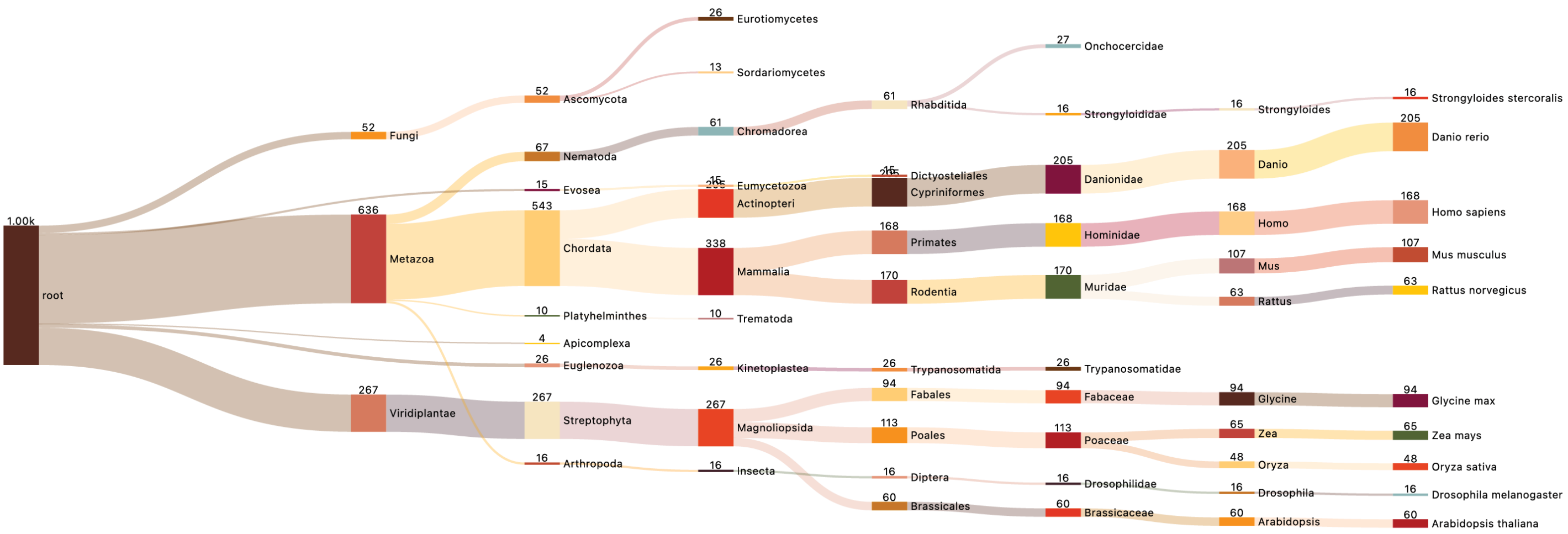

TRIM8 RING (1-81) top 25 AlphaFold Database Proteome hits by E-Value

Target	Description	Scientific Name	Prob.	Seq. Id.	E-Value	Start	End
AF-Q9BZR9-F1-model_v6	E3 ubiquitin-protein ligase TRIM8	Homo sapiens	1	100	3.51E-16	1	81
AF-D3ZQE3-F1-model_v6	E3 ubiquitin-protein ligase TRIM8	Rattus norvegicus	1	100	2.33E-15	1	81
AF-Q99PJ2-F1-model_v6	E3 ubiquitin-protein ligase TRIM8	Mus musculus	1	100	3.49E-15	1	81
AF-J9UF94-F1-model_v6	E3 ubiquitin-protein ligase TRIM8	Danio rerio	1	86.2	4.56E-14	1	81
AF-B7ZVP9-F1-model_v6	E3 ubiquitin-protein ligase TRIM8b	Danio rerio	1	83.7	1.10E-13	1	81
AF-A0A8M2B489-F1-model_v6	Bloodthirsty-related gene family, member 16 isoform X1	Danio rerio	1	40.4	7.68E-09	1	81
AF-I3ISK5-F1-model_v6	Bloodthirsty-related gene family, member 18	Danio rerio	1	41.8	2.77E-08	7	81
AF-A0A8M2BAS0-F1-model_v6	E3 ubiquitin/ISG15 ligase TRIM25	Danio rerio	1	34.1	3.40E-08	1	81
AF-A0AB13AA62-F1-model_v6	Bloodthirsty-related gene family, member 22	Danio rerio	1	41.6	5.10E-08	9	81
AF-A0AB32T7E1-F1-model_v6	E3 ubiquitin-protein ligase TRIM39	Danio rerio	1	34.6	6.24E-08	6	81
AF-A0A1D5NS27-F1-model_v6	Bloodthirsty-related gene family, member 4	Danio rerio	1	40.2	7.15E-08	9	81
AF-A0A0R4IUT0-F1-model_v6	Bloodthirsty-related gene family, member 20	Danio rerio	1	41.6	8.18E-08	9	81
AF-A9JSZ8-F1-model_v6	Bloodthirsty-related gene family, member 25	Danio rerio	1	40.2	8.18E-08	9	81
AF-A0AB32T9P6-F1-model_v6	E3 ubiquitin-protein ligase TRIM39-like	Danio rerio	1	41.6	8.75E-08	9	81
AF-A0AB32T677-F1-model_v6	E3 ubiquitin/ISG15 ligase TRIM25 isoform X1	Danio rerio	1	40.8	8.75E-08	10	81
AF-A0A8N7T6I4-F1-model_v6	Tripartite motif-containing protein 16	Danio rerio	1	39.7	9.37E-08	10	81
AF-A0A8M9PXG9-F1-model_v6	E3 ubiquitin/ISG15 ligase TRIM25	Danio rerio	1	38.8	9.37E-08	10	81
AF-A0A1D5NS95-F1-model_v6	Bloodthirsty-related gene family, member 7	Danio rerio	1	34.6	9.37E-08	3	81
AF-A0A0R4IEH8-F1-model_v6	E3 ubiquitin-protein ligase TRIM39	Danio rerio	1	40.5	1.00E-07	7	81
AF-A0A8M3B540-F1-model_v6	RING-type domain-containing protein	Danio rerio	1	36.6	1.00E-07	10	81
AF-A0A8N7TDQ8-F1-model_v6	Tripartite motif-containing protein 16	Danio rerio	1	35.6	1.00E-07	10	81
AF-A0A8M1RMR4-F1-model_v6	E3 ubiquitin-protein ligase TRIM39	Danio rerio	1	42.2	1.07E-07	9	79
AF-Q1RLP5-F1-model_v6	Bloodthirsty-related gene family, member 32	Danio rerio	1	40	1.07E-07	1	80
AF-Q14258-F1-model_v6	E3 ubiquitin/ISG15 ligase TRIM25	Homo sapiens	1	46.5	1.15E-07	8	80
AF-M0R947-F1-model_v6	E3 ubiquitin-protein ligase TRIM62	Rattus norvegicus	1	44.5	1.15E-07	10	81

Figure S3 – FoldSeek analysis of TRIM8 RING domain

A-B, TRIM8 RING domain (residues 1-81) were used as search input on the FoldSeek tertiary structure alignment tool to interrogate the PDB (**A**) or the AlphaFold database (**B**), with the top 25 hits from each provided as a table.

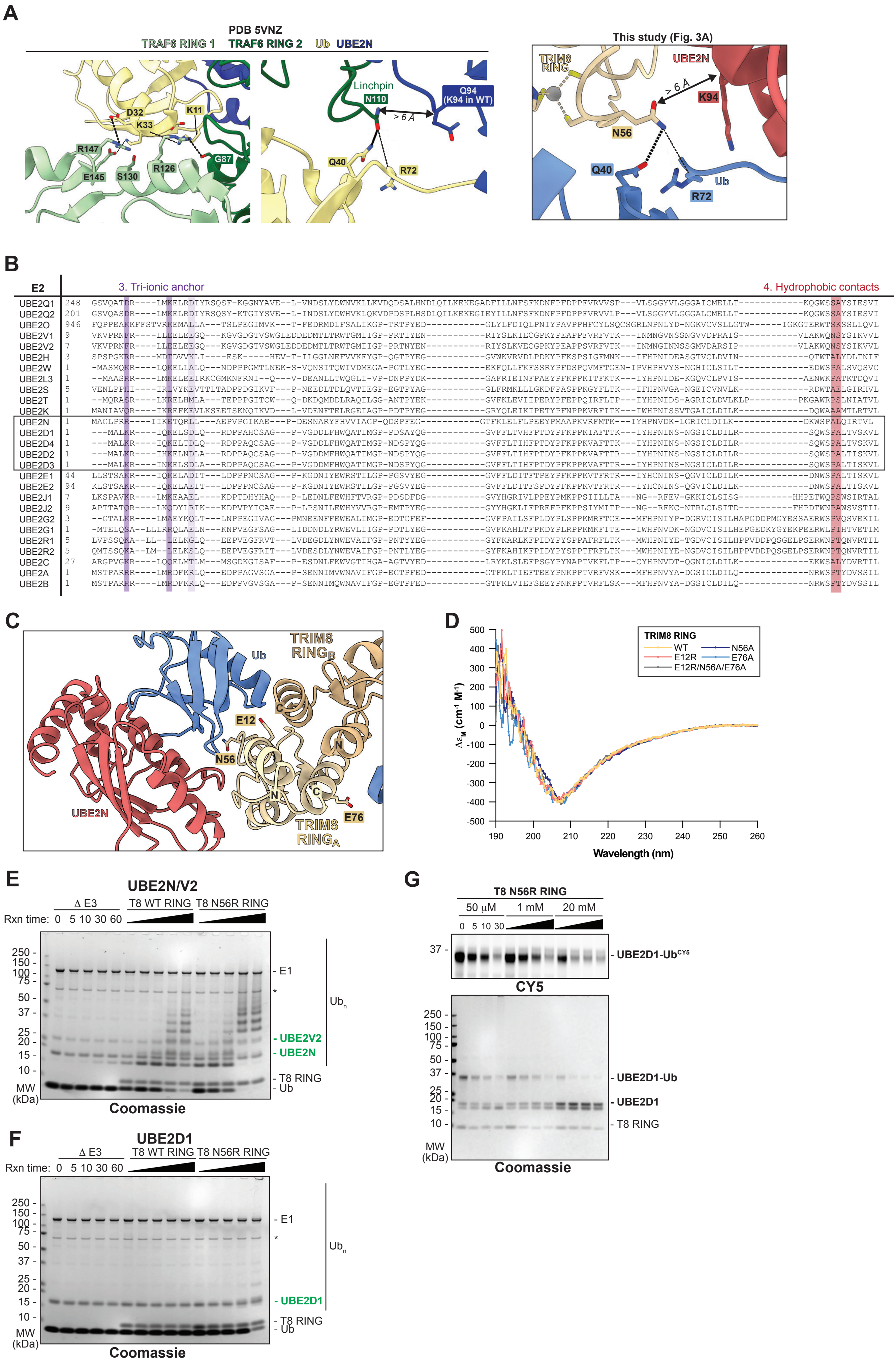
A
PDB 5VNZ
TRAF6 RING 1
TRAF6 RING 2
Ub
UBE2N
D32
K11
K33
R147
R126
G87
E145
S130
Linchpin
N110
Q94
(K94 in WT)
> 6 Å
Q40
R72
This study (Fig. 3A)
TRIM8 RING
UBE2N
> 6 Å
K94
N56
Q40
Ub
R72
B
E2
3. Tri-ionic anchor
4. Hydrophobic contacts
UBE2Q1
UBE2Q2
UBE2O
UBE2V1
UBE2V2
UBE2H
UBE2W
UBE2L3
UBE2S
UBE2T
UBE2K
UBE2N
UBE2D1
UBE2D4
UBE2D2
UBE2D3
UBE2E1
UBE2E2
UBE2J1
UBE2J2
UBE2G2
UBE2G1
UBE2R1
UBE2R2
UBE2C
UBE2A
UBE2B
C
Ub
TRIM8 RING_B
E12
N56
UBE2N
TRIM8 RING_A
E76
D
TRIM8 RING
WT
N56A
E12R
E76A
E12R/N56A/E76A
Δε_M (cm⁻¹ M⁻¹)
Wavelength (nm)
E
UBE2N/V2
Δ E3
T8 WT RING
T8 N56R RING
Rxn time: 0 5 10 30 60
250
150
100
75
50
37
25
20
15
10
MW (kDa)
E1
*
Ub_n
UBE2V2
UBE2N
T8 RING
Ub
Coomassie
F
UBE2D1
Δ E3
T8 WT RING
T8 N56R RING
Rxn time: 0 5 10 30 60
E1
*
Ub_n
UBE2D1
T8 RING
Ub
MW (kDa)
Coomassie
G
T8 N56R RING
50 μM
1 mM
20 mM
0 5 10 30
37
UBE2D1-Ub^CY5
CY5
UBE2D1-Ub
UBE2D1
T8 RING
MW (kDa)
Coomassie

Figure S4 – Data exploring the significance of TRIM8 asparagine linchpin residue

A, Cartoon representations of TRAF6/UBE2N-Ub crystal structure (PDB 5VNZ (41): TRAF6 shown in light and dark green, UBE2N in dark blue, and ubiquitin in yellow) and TRIM8/UBE2N-Ub structure (reproduction from Fig. 3A for comparison), with key interacting residues labelled. **B**, Multiple sequence alignment of human E2s, with residues corresponding UBE2N-TRIM8 contacts highlighted (see Fig. 3). **C**, Cartoon representation of the crystal structure of the TRIM8 RING/UBE2N-Ub complex (see Fig. 3) with E12, N56, and E76 residues of $RING_A$ shown as sticks. **D**, Circular dichroism (CD) spectra of TRIM8 WT and mutant RING domains CD spectra at 25 °C were recorded between 190 and 160 nm. Delta epsilon molar units ($\Delta\varepsilon$) normalised spectra are shown. **E-F**, Coomassie gel scans of SDS-PAGE gels of ubiquitination reactions carried out at 30°C using 2 µM UBE2N/V2 (**D**) or UBE2D1 (**E**), 50 µM ubiquitin, 1 µM UBA1, 1 µM Ub^{ATTO} , and 3 mM ATP, alongside 2 µM wild-type (WT) or N56R mutant TRIM8 RING domains (n = 3, see also **Fig. 4C-D**). **G**, Fluorescent (Ub^{CY5}) and Coomassie gel scans showing the discharge of Ub^{CY5} from pre-charged UBE2D1 onto 1 mM free lysine in solution over time at 25°C, with 2 µM WT or N56R TRIM8 RING or TRIM21 RING (see **Fig. 4B**).

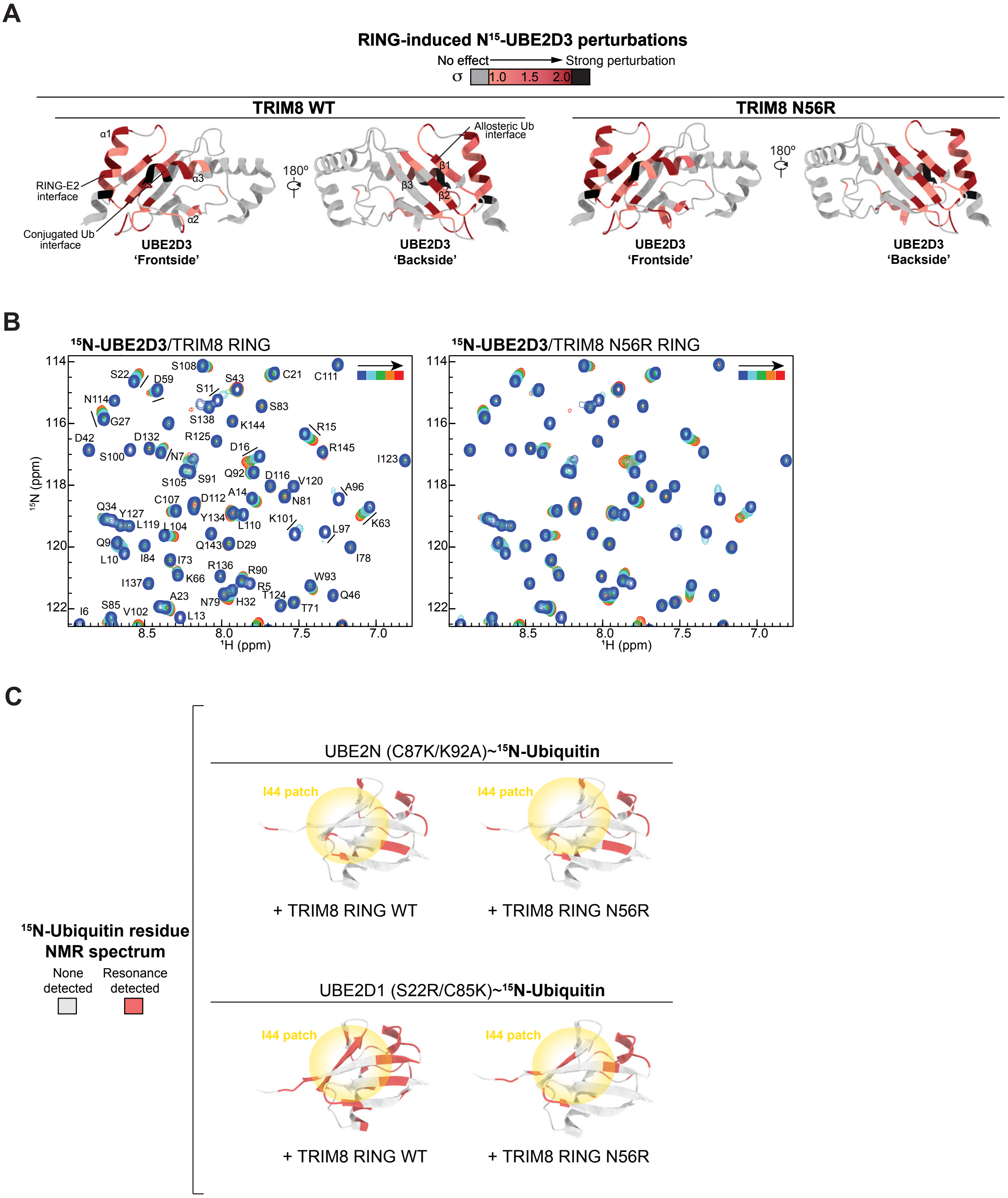
A
RING-induced N15-UBE2D3 perturbations
No effect
Strong perturbation
σ
1.0 1.5 2.0
TRIM8 WT
TRIM8 N56R
α1
α2
α3
RING-E2 interface
Conjugated Ub interface
Allosteric Ub interface
β1
β2
β3
180°
UBE2D3 'Frontside'
UBE2D3 'Backside'
B
15N-UBE2D3/TRIM8 RING
15N-UBE2D3/TRIM8 N56R RING
15N (ppm)
1H (ppm)
114
116
118
120
122
8.5
8.0
7.5
7.0
C
UBE2N (C87K/K92A)~15N-Ubiquitin
UBE2D1 (S22R/C85K)~15N-Ubiquitin
I44 patch
+ TRIM8 RING WT
+ TRIM8 RING N56R
15N-Ubiquitin residue NMR spectrum
None detected
Resonance detected

Figure S5 – NMR analyses of perturbation of ^{15}N-labelled UBE2D3 or ubiquitin by TRIM8 WT or N56R mutant

A-B, ^{15}N-labelled UBE2D3 S22R ^{1}H/^{15}N chemical shift perturbations induced by the presence of WT (left) or N56R mutant (right) TRIM8 RING domain: **A**, Mapped onto the structure of UBE2D3 (PDB 5EGG (64)), coloured with red gradient to indicate degree of chemical shift (multiples of σ) or black where the signal is attenuated; **B**, Details of the ^{1}H-^{15}N HSQC spectra, with blue to red at different ligand concentrations (0, 75, 150, 225, 300 μM) plotted at the same contour level. **C**, ^{15}N-labelled ubiquitin ^{1}H/^{15}N resonances in UBE2D1-Ub or UBE2N-Ub conjugates with or without WT or N56R mutant TRIM8 RING domains, mapped onto the structure of ubiquitin (PDB 1UBQ (63)) (coloured red where resonances have been detected and light grey where resonances are not detected), with the I44 hydrophobic patch highlighted.

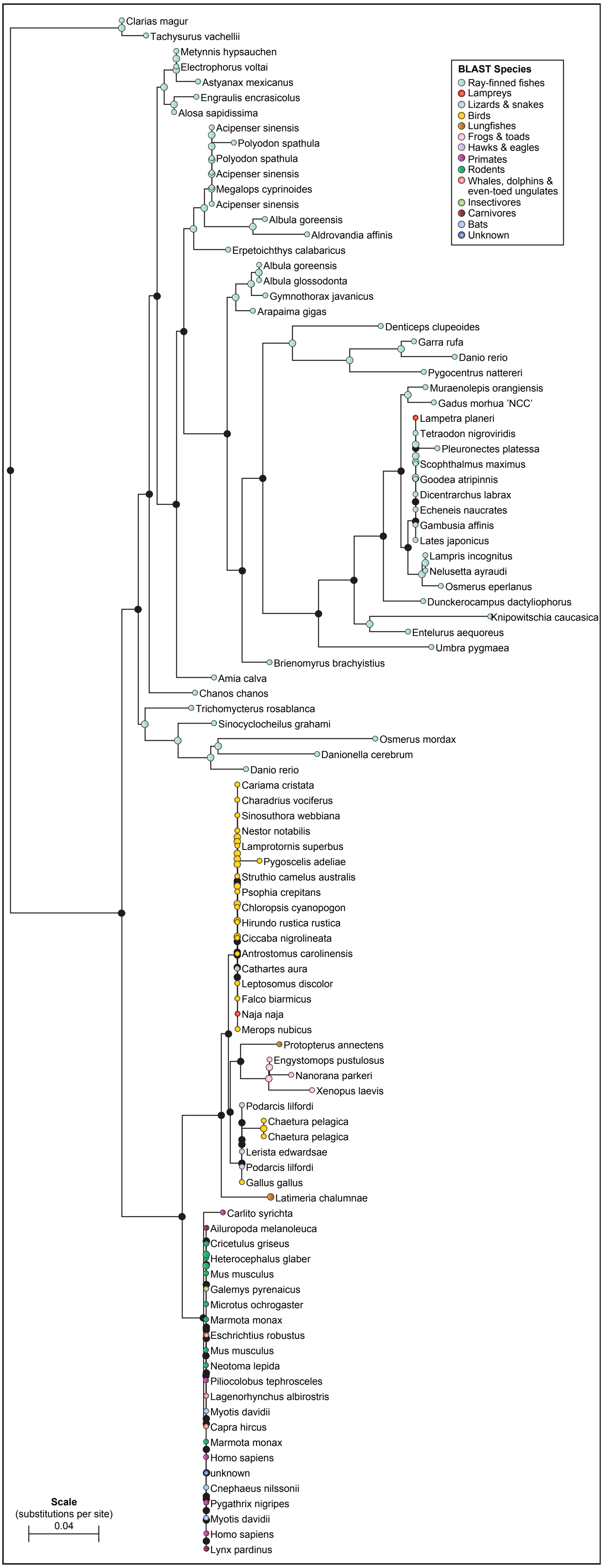
Clarias magur
Tachysurus vachellii
Metynnis hypsauchen
Electrophorus voltai
Astyanax mexicanus
Engraulis encrasicolus
Alosa sapidissima
Acipenser sinensis
Polyodon spathula
Polyodon spathula
Acipenser sinensis
Megalops cyprinoides
Acipenser sinensis
Albula goreensis
Aldrovandia affinis
Erpetoichthys calabaricus
Albula goreensis
Albula glossodonta
Gymnothorax javanicus
Arapaima gigas
Denticeps clupeoides
Garra rufa
Danio rerio
Pygocentrus nattereri
Muraenolepis orangiensis
Gadus morhua 'NCC'
Lampetra planeri
Tetraodon nigroviridis
Pleuronectes platessa
Scophthalmus maximus
Goodea atripinnis
Dicentrarchus labrax
Echeneis naucrates
Gambusia affinis
Lates japonicus
Lampris incognitus
Nelusetta ayraudi
Osmerus eperlanus
Dunckerocampus dactyliophorus
Knipowitschia caucasica
Entelurus aequoreus
Umbra pygmaea
Brienomyrus brachyistius
Amia calva
Chanos chanos
Trichomycterus rosablanca
Sinocyclocheilus grahami
Osmerus mordax
Danionella cerebrum
Danio rerio
Cariama cristata
Charadrius vociferus
Sinosuthora webbiana
Nestor notabilis
Lamprotornis superbus
Pygoscelis adeliae
Struthio camelus australis
Psophia crepitans
Chloropsis cyanopogon
Hirundo rustica rustica
Ciccaba nigrolineata
Antrostomus carolinensis
Cathartes aura
Leptosomus discolor
Falco biarmicus
Naja naja
Merops nubicus
Protopterus annectens
Engystomops pustulosus
Nanorana parkeri
Xenopus laevis
Podarcis lilfordi
Chaetura pelagica
Chaetura pelagica
Lerista edwardsae
Podarcis lilfordi
Gallus gallus
Latimeria chalumnae
Carlito syrichta
Ailuropoda melanoleuca
Cricetulus griseus
Heterocephalus glaber
Mus musculus
Galemys pyrenaicus
Microtus ochrogaster
Marmota monax
Eschrichtius robustus
Mus musculus
Neotoma lepida
Piliocolobus tephrosceles
Lagenorhynchus albirostris
Myotis davidii
Capra hircus
Marmota monax
Homo sapiens
unknown
Cnephaeus nilssonii
Pygathrix nigripes
Myotis davidii
Homo sapiens
Lynx pardinus
BLAST Species
Ray-finned fishes
Lampreys
Lizards & snakes
Birds
Lungfishes
Frogs & toads
Hawks & eagles
Primates
Rodents
Whales, dolphins & even-toed ungulates
Insectivores
Carnivores
Bats
Unknown
Scale
(substitutions per site)
0.04

Figure S6 – TRIM8 RING protein sequence conservation in metazoans

Neighbour joining tree of ClusteredNR BLAST protein sequence alignment results for TRIM8 RING domain (residues 1-81), with all clusters expanded, species colour coded, and taxonomic names used as labels (where available).

Supplementary Table 1

X-ray crystallography statistics

	**TRIM8 RING/UBE2N~Ub**
Wavelength (Å)	0.9537
Resolution range (Å)	49 - 2.39 (2.47 - 2.39)
Space group	P 1 21 1
Cell dimensions	
a, b, c (Å)	49.5 177.5 75.6
α, β, γ (°)	90 97.8 90
Total reflections	317705 (26526)
Unique reflections	50697 (4566)
Multiplicity	6.3 (5.8)
Completeness (%)	99.7 (96.6)
Mean I/sigma(I)	6.2 (0.9)
Wilson B-factor	51.5
R-meas	0.193 (2.4)
R-pim	0.076 (0.98)
CC1/2	0.98 (0.36)
CC*	0.99 (0.66)
Reflections used in refinement	50582 (2516)
Reflections used for R-free	2605 (133)
R-work	0.201 (0.316)
R-free	0.253 (0.356)
CC (work)	
CC (free)	
Number of non-hydrogen atoms	9879
Macromolecules	9693
Ligands	8
Solvent	178
Protein residues	1221
RMS (bonds) (Å)	0.004
RMS (angles) (°)	0.84
Ramachandran favored (%)	98.08
Ramachandran allowed (%)	1.92
Ramachandran outliers (%)	0.00
Rotamer outliers (%)	0.38
Clashscore	6.98
Average B-factor ($Å^2$)	58.44
Macromolecules ($Å^2$)	58.55
Ligands ($Å^2$)	46.96
Solvent ($Å^2$)	52.84

Supplementary Table 2

ClusteredNR BLAST protein sequence alignments for TRIM8 RING domain (residues 1-81)

Protein name	Species	Sequence ID	Sequence
tripartite motif containing 8	*Homo sapiens*	gb|KAI2557100.1|/1-76	MAENWKNCFE-------EELICPICLHVFVEPVQLPCKHNFCRGCIGEAWAKDSGLVRCPEC**N**QAYNQKPGLEKNLKLTNIVE
Hypothetical predicted protein	*Marmota monax*	emb|VTJ76969.1|/1-76	MAENWKNCFE-------EELICPICLHVFVEPVQLPCKHNFCRGCIGEAWAKDSGLVRCPEC**N**QAYNQKPGLEKNLKLTNIVE
hypothetical protein GHT09_020385	*Marmota monax*	gb|KAF7459632.1|/1-76	MAENWKNCFE-------EELICPICLHVFVEPVQLPCKHNFCRGCIGEAWAKDSGLVRCPEC**N**QAYNQKPGLEKNLKLTNIVE
hypothetical protein A6R68_01956	*Neotoma lepida*	gb|OBS69519.1|/1-76	MAENWKNCFE-------EELICPICLHVFVEPVQLPCKHNFCRGCIGEAWAKDSGLVRCPEC**N**QAYNQKPGLEKNLKLTNIVE
putative E3 ubiquitin-protein ligase TRIM8	*Cricetulus griseus*	gb|ERE79579.1|/1-76	MAENWKNCFE-------EELICPICLHVFVEPVQLPCKHNFCRGCIGEAWAKDSGLVRCPEC**N**QAYNQKPGLEKNLKLTNIVE
putative E3 ubiquitin-protein ligase TRIM8, partial	*Eschrichtius robustus*	gb|MBW01606.1|/1-76	MAENWKNCFE-------EELICPICLHVFVEPVQLPCKHNFCRGCIGEAWAKDSGLVRCPEC**N**QAYNQKPGLEKNLKLTNIVE
hypothetical protein H8959_011110	*Pygathrix nigripes*	gb|KAL4698453.1|/1-76	MAENWKNCFE-------EELICPICLHVFVEPVQLPCKHNFCRGCIGEAWAKDSGLVRCPEC**N**QAYNQKPGLEKNLKLTNIVE
probable E3 ubiquitin-protein ligase TRIM8, partial	*Carlito syrichta*	ref|XP_021564982.1|/1-76	MAENWKNCFE-------EELICPICLHVFVEPVQLPCKHNFCRGCIGEAWAKDSGLVRCPEC**N**QAYNQKPGLEKNLKLANIVE
E3 ubiquitin-protein ligase TRIM8 isoform X2	*Piliocolobus tephrosceles*	ref|XP_023062284.1|/1-76	MAENWKNCFE-------EELICPICLHVFVEPVQLPCKHNFCRGCIGEAWAKDSGLVRCPEC**N**QAYNQKPGLEKNLKLTNIVE
Trim8 protein, partial	*Mus musculus*	gb|AAH37065.1|/1-76	MAENWKNCFE-------EELICPICLHVFVEPVQLPCKHNFCRGCIGEAWAKDSGLVRCPEC**N**QAYNQKPGLEKNLKLTNIVE
PREDICTED: probable E3 ubiquitin-protein ligase TRIM8	*Myotis davidii*	ref|XP_015414756.1|/1-76	MAENWKNCFE-------EELICPICLHVFVEPVQLPCKHNFCRGCIGEAWAKDSGLVRCPEC**N**QAYNQKPGLEKNLKLTNIVE
low quality protein: tripartite	*Lynx pardinus*	emb|VFV24422.1|/1-76	MAENWKNCFE-------EELICPICLHVFVEPVQLPCKHNFCRGCIGEAWAKDSGLVRCPEC**N**QAYNQKPGLEKNLKLTNIVE
E3 ubiquitin-protein ligase TRIM8 isoform 2	*Homo sapiens*	ref|NP_001332879.1|/1-76	MAENWKNCFE-------EELICPICLHVFVEPVQLPCKHNFCRGCIGEAWAKDSGLVRCPEC**N**QAYNQKPGLEKNLKLTNIVE
hypothetical protein K5549_012499	*Capra hircus*	gb|KAJ1067140.1|/1-76	MAENWKNCFE-------EELICPICLHVFVEPVQLPCKHNFCRGCIGEAWAKDSGLVRCPEC**N**QAYNQKPGLEKNLKLTNIVE
E3 ubiquitin-protein ligase TRIM8 isoform X1	*Ailuropoda melanoleuca*	ref|XP_034519086.1|/1-76	MAENWKNCFE-------EELICPICLHVFVEPVQLPCKHNFCRGCIGEAWAKDSGLVRCPEC**N**QAYNQKPGLEKNLKLTNIVE
E3 ubiquitin-protein ligase TRIM8 isoform 1	*Mus musculus*	ref|NP_444330.2|/1-76	MAENWKNCFE-------EELICPICLHVFVEPVQLPCKHNFCRGCIGEAWAKDSGLVRCPEC**N**QAYNQKPGLEKNLKLTNIVE
hypothetical protein QTO34_005891, partial	*Cnephaeus nilssonii*	gb|KAK1333507.1|/73-148	MAENWKNCFE-------EELICPICLHVFVEPVQLPCKHNFCRGCIGEAWAKDSGLVRCPEC**N**QAYNQKPGLEKNLKLTNIVE
Tripartite motif-containing protein 8	*Heterocephalus glaber*	gb|EHB18540.1|/1-76	MAENWKNCFE-------EELICPICLHVFVEPVQLPCKHNFCRGCIGEAWAKDSGLVRCPEC**N**QAYNQKPGLEKNLKLTNIVE
E3 ubiquitin-protein ligase TRIM8 isoform X1	*Lagenorhynchus albirostris*	ref|XP_059981683.1|/1-76	MAENWKNCFE-------EELICPICLHVFVEPVQLPCKHNFCRGCIGEAWAKDSGLVRCPEC**N**QAYNQKPGLEKNLKLTNIVE
putative E3 ubiquitin-protein ligase TRIM8	*Microtus ochrogaster*	gb|KAH0504556.1|/124-199	MAENWKNCFE-------EELICPICLHVFVEPVQLPCKHNFCRGCIGEAWAKDSGLVRCPEC**N**QAYNQKPGLEKNLKLTNIVE
E3 ubiquitin-protein ligase TRIM8	*Galemys pyrenaicus*	gb|KAG8523596.1|/24-99	MAENWKNCFE-------EELICPICLHVFVEPVQLPCKHNFCRGCIGEAWAKDSGLVRCPEC**N**QAYNQKPGLEKNLKLTNIVE
TRIM8 ligase, partial	*Paradoxornis webbianus*	gb|NWR06881.1|/1-76	MAENWKNCFE-------EELICPICLHVFVEPVQLPCKHNFCRGCIGEAWAKESGLVRCPEC**N**QAYNQKPNLEKNLKLTNIVE
PREDICTED: probable E3 ubiquitin-protein ligase TRIM8, partial	*Cariama cristata*	ref|XP_009697056.1|/1-76	MAENWKNCFE-------EELICPICLHVFVEPVQLPCKHNFCRGCIGEAWAKESGLVRCPEC**N**QAYNQKPNLEKNLKLTNIVE
TRIM8 ligase, partial	*Psophia crepitans*	gb|NXI93559.1|/1-76	MAENWKNCFE-------EELICPICLHVFVEPVQLPCKHNFCRGCIGEAWAKESGLVRCPEC**N**QAYNQKPNLEKNLKLTNIVE
PREDICTED: probable E3 ubiquitin-protein ligase TRIM8	*Charadrius vociferus*	ref|XP_009879913.1|/1-76	MAENWKNCFE-------EELICPICLHVFVEPVQLPCKHNFCRGCIGEAWAKESGLVRCPEC**N**QAYNQKPNLEKNLKLTNIVE
PREDICTED: probable E3 ubiquitin-protein ligase TRIM8, partial	*Merops nubicus*	ref|XP_008948412.1|/1-76	MAENWKNCFE-------EELICPICLHVFVEPVQLPCKHNFCRGCIGEAWAKESGLVRCPEC**N**QAYNQKPNLEKNLKLTNIVE
PREDICTED: probable E3 ubiquitin-protein ligase TRIM8, partial	*Leptosomus discolor*	ref|XP_009948779.1|/1-76	MAENWKNCFE-------EELICPICLHVFVEPVQLPCKHNFCRGCIGEAWAKESGLVRCPEC**N**QAYNQKPNLEKNLKLTNIVE
putative E3 ubiquitin-protein ligase TRIM8	*Struthio camelus australis*	gb|KFV76043.1|/1-76	MAENWKNCFE-------EELICPICLHVFVEPVQLPCKHNFCRGCIGEAWAKESGLVRCPEC**N**QAYNQKPNLEKNLKLTNIVE
putative E3 ubiquitin-protein ligase TRIM8	*Cathartes aura*	gb|KFP50914.1|/1-76	MAENWKNCFE-------EELICPICLHVFVEPVQLPCKHNFCRGCIGEAWAKESGLVRCPEC**N**QAYNQKPNLEKNLKLTNIVE
PREDICTED: probable E3 ubiquitin-protein ligase TRIM8	*Chaetura pelagica*	ref|XP_009998467.1|/1-76	MAENWKNCFE-------EELICPICLHVFVEPVQLPCKHNFCRGCIGEAWAKETGLVRCPEC**N**QAYNQKPNLEKILKLTNIVE
Putative E3 ubiquitin-protein ligase TRIM8	*Myotis davidii*	gb|ELK35608.1|/1-76	MAENWKNCFE-------EELICPICLHVFVEPVQLPCKHNFCRGCIGEAWAKDSGLVRCPEC**N**QAYNQKPGLEKNLKLTNIVE
LOW QUALITY PROTEIN: E3 ubiquitin-protein ligase TRIM8	*Antrostomus carolinensis*	ref|XP_028940755.1|/1-76	MAENWKNCFE-------EELICPICLHVFVEPVQLPCKHNFCRGCIGEAWAKESGLVRCPEC**N**QAYNQKPNLEKNLKLTNIVE
hypothetical protein IHE44_0001520, partial	*Lamprotornis superbus*	gb|KAI1236241.1|/53-128	MAENWKNCFE-------EELICPICLHVFVEPVQLPCKHNFCRGCIGEAWAKESGLVRCPEC**N**QAYNQKPNLEKNLKLTNIVE
PREDICTED: probable E3 ubiquitin-protein ligase TRIM8, partial	*Nestor notabilis*	ref|XP_010011741.1|/1-76	MAENWKNCFE-------EELICPICLHVFVEPVQLPCKHNFCRGCIGEAWAKESGLVRCPEC**N**QAYNQKPNLEKNLKLTNIVE
TRIM8 ligase, partial	*Ciccaba nigrolineata*	gb|NXF64113.1|/1-76	MAENWKNCFE-------EELICPICLHVFVEPVQLPCKHNFCRGCIGEAWAKESGLVRCPEC**N**QAYNQKPNLEKNLKLTNIVE
putative E3 ubiquitin-protein	*Naja naja*	gb|KAG8135690.1|/1-76	MAENWKNCFE-------EELICPICLHVFVEPVQLPCKHNFCRGCIGEAWAKESGLVRCPEC**N**QAYNQKPNLEKNLKLTNIVE
hypothetical protein GDO81_004175	*Engystomops pustulosus*	gb|KAG8551590.1|/1-76	MAENWKNCFE-------EELICPICLHVFIEPVQLPCKHNFCRGCISEAWAKETGLVRCPEC**N**QAYNQKPNLEKNLKLTNIVE
hypothetical protein lerEdw1_014303	*Lerista edwardsae*	gb|KAJ6633773.1|/1-76	MAENWKNCFE-------EELICPICLHVFVEPVQLPCKHNFCRGCIGEAWAKETGLVRCPEC**N**QAYNQKPNLEKNLKLTNIVE
E3 ubiquitin-protein ligase TRIM8	*Protopterus annectens*	ref|XP_043912480.1|/1-76	MAENWKNCFE-------EELICPICLHVFIEPVQLPCKHNFCRGCIGEAWAKESGLVRCPEC**N**QAYNQKPNLEKNLKLTNIV**D**
E3 ubiquitin-protein ligase TRIM8	*Podarcis lilfordi*	emb|CAI5775125.1|/1-76	MAENWKNCFE-------EELICPICLHVFVEPVQLPCKHNFCRGCIGEAWAKETGLVRCPEC**N**QAYNQKPNLEKNLKLTNIVE
E3 ubiquitin-protein ligase TRIM8	*Gallus gallus*	ref|NP_001026404.2|/1-76	MAENWKNCFE-------EELICPICLHVFVEPVQLPCKHNFCRGCIGEAWAKETGLVRCPEC**N**QAYNQKPNLEKNLKLTNIVE
hypothetical protein DUI87_11561	*Hirundo rustica rustica*	gb|RMC11442.1|/1-76	MAENWKNCFE-------EELICPICLHVFVEPVQLPCKHNFCRGCIGEAWAKESGLVRCPEC**N**QAYNQKPNLEKNLKLTNIVE
PREDICTED: probable E3 ubiquitin-protein ligase TRIM8	*Pygoscelis adeliae*	ref|XP_009321552.1|/1-76	MAENWKNCFE-------EEVICPICLHVFVEPVQLPCKHNFCRGCIGEAWAKESGLVRCPEC**N**QAYNQKPNLEKNLKLTNIVE
E3 ubiquitin-protein ligase TRIM8	*Podarcis lilfordi*	emb|CAI5775123.1|/1-76	MAENWKNCFE-------EELICPICLHVFVEPVQLPCKHNFCRGCIGEAWAKETGLVRCPEC**N**QAYNQKPNLEKNLKLTNIVE
TRIM8 ligase, partial	*Chloropsis cyanopogon*	gb|NXP57818.1|/1-76	MAENWKNCFE-------EELICPICLHVFVEPVQLPCKHNFCRGCIGEAWAKESGLVRCPEC**N**QAYNQKPNLEKNLKLTNIVE
E3 ubiquitin-protein ligase TRIM8a	*Engraulis encrasicolus*	ref|XP_063047115.1|/1-76	MAENWKNCFE-------EELICPICLHVFVEPIQLPCKHNFCRGCISEAWAKDTGMVRCPEC**N**HAYNQKPNLEKNIKLTNIVE
E3 ubiquitin-protein ligase TRIM8 isoform X1	*Falco biarmicus*	ref|XP_056207417.1|/140-215	MAENWKNCFE-------EELICPICLHVFVEPVQLPCKHNFCRGCIGEAWAKESGLVRCPEC**N**QAYNQKPNLEKNLKLTNIVE
E3 ubiquitin-protein ligase TRIM8b	*Amia ocellicauda*	ref|XP_066549128.1|/1-76	MAENWKNCFE-------EELICPICLHVFVEPIQLPCKHNFCRGCISEAWAKDAGMVRCPEC**N**HAYNQKPSLEKNLKLTNIVE
hypothetical protein FKN15_070029	*Acipenser sinensis*	gb|MGH0136622.1|/1-76	MAENWKNCFE-------EELICPICLHVFVEPIQLPCKHNFCRGCISEAWSKDTGMVRCPEC**N**HAYNQKPTLEKNLKLTNIVE
putative E3 ubiquitin-protein ligase TRIM8	*Chaetura pelagica*	gb|KFU91522.1|/1-76	MAENWKNCFE-------EELICPICLHVFVEPVQLPCKHNFCRGCIGEAWAKETGLVRCPEC**N**QAYNQKPNLEKILKLTNIVE

TRIM8 ligase, partial	*Polyodon spathula*	gb|MBN3284625.1|/1-76	MAENWKNCFE-------EELICPICLHVFVEPIQLPCKHNFCRGCISEAWSKDTGMVRCPEC**N**HAYNQKPTLEKNLKLTNIVE
hypothetical protein MHYP_G00040170	*Metynnis hypsauchen*	gb|KAL6487391.1|/8-83	MAENWKNCFE-------EELICPICLHVFVEPIQLPCKHNFCRGCISEAWAKDTGLVRCPEC**N**HAYNQKPNLEKNIKLTNIVE
E3 ubiquitin-protein ligase TRIM8b	*Latimeria chalumnae*	ref|XP_006007437.1|/1-76	MAENWKSCFE-------EELICPICLHVFVEPVQLPCKHNFCRGCIGEAWAKEAGLVRCPEC**N**QAYNQKPSLEKNLKLTNIVE
PREDICTED: probable E3 ubiquitin-protein ligase TRIM8 isoform X2	*Nanorana parkeri*	ref|XP_018408925.1|/1-76	MAENWKNCFE-------EELICPICLHVFIEPVQLPCKHNFCRGCISEAWAKETGLVRCPEC**N**QAYNQKPNLEKNLKLTNIVE
E3 ubiquitin-protein ligase TRIM8a	*Sinocyclocheilus grahami*	ref|XP_016110997.1|/8-83	MAENWRNCFE-------EELICPICLHVFVEPVQLPCKHNFCRGCISEAWAKDTANVRCPEC**N**HAYNQKPNLEKNIKLTNIVE
E3 ubiquitin-protein ligase TRIM8a isoform X2	*Chanos chanos*	ref|XP_030628518.1|/1-76	MAEDWKNCFE-------EELICPICLHVFVEPIQLPCKHNFCRGCISEAWAKDSGIVRCPEC**N**HAYNQKPNLEKNIKLTNIVE
E3 ubiquitin-protein ligase TRIM8-like	*Alosa pseudoharengus*	ref|XP_076142587.1|/1-76	MAENWKNCFE-------EELICPICLHVFVEPIQLPCKHNFCRGCISEAWAKDTGMVRCPEC**N**HAYNQKPNLEKNIKLTNIVE
E3 ubiquitin-protein ligase TRIM8a	*Alosa sapidissima*	ref|XP_041922642.1|/1-76	MAENWKNCFE-------EELICPICLHVFVEPIQLPCKHNFCRGCISEAWAKDTGMVRCPEC**N**HAYNQKPNLEKNIKLTNIVE
hypothetical protein P4O66_013529	*Electrophorus voltai*	gb|KAK1791524.1|/8-83	MAENWKNCFE-------EELICPICLHVFVEPIQLPCKHNFCRGCISEAWAKDTGLVRCPEC**N**HAYNQKPNLEKNIKLTNIVE
hypothetical protein JZ751_007979	*Albula glossodonta*	gb|KAG9346161.1|/1-76	MAENWKNCFE-------EELICPICLHVFTEPIQLPCKHNFCRGCISEAWSKDTGMVRCPEC**N**HAYNQKPSLEKNHKLSNIVE
hypothetical protein GJAV_G00150320	*Gymnothorax javanicus*	gb|KAJ8264527.1|/1-76	MAENWKNCFE-------EELICPICLHVFTEPIQLPCKHNFCRGCISEAWSKDTGIVRCPEC**N**HAYNQKPSLEKNHKLSNIVE
hypothetical protein AGOR_G00202060	*Albula goreensis*	gb|KAI1887052.1|/1-76	MAENWKNCFE-------EELICPICLHVFTEPIQLPCKHNFCRGCISEAWSKDTGMVRCPEC**N**HAYNQKPSLEKNHKLSNIVE
E3 ubiquitin-protein ligase TRIM8-like	*Megalops cyprinoides*	ref|XP_036403224.1|/1-76	MAENWKNCFE-------EELICPICLHVFVEPIQLPCKHNFCRGCISEAWSKDTGMVRCPEC**N**HAYNQKPTLEKNLKLTNIVE
tripartite motif containing 8 L homeolog	*Xenopus laevis*	ref|NP_001106307.1|/1-76	MAENWKNCFE-------EELICPICLHVFIEPVQLPCKHNFCRGCISEAWAKETGMVRCPEC**N**QAYNQKPTLEKNLKLTNIVE
E3 ubiquitin-protein ligase TRIM8-like	*Erpetoichthys calabaricus*	ref|XP_028651569.1|/1-76	MAENWKNCFE-------EELICPICLHVFVEPIQLPCKHNFCRGCISEAWSKDTGLVRCPEC**N**HAYNQKPVLEKNLKLTNIVE
hypothetical protein FKN15_030164	*Acipenser sinensis*	gb|MGH0128575.1|/1-76	MAENWKNCFE-------EELICPICLHVFVEPIQLPCKHNFCRGCISEAWSKDTGMVRCPEC**N**HAYNQKPTLEKNLKLTNIVE
E3 ubiquitin-protein ligase TRIM8a	*Astyanax mexicanus*	ref|XP_007235869.1|/8-83	MAENWKNCFE-------EELICPICLHVFVEPIQLPCKHNFCRGCISEAWAKETGLVRCPEC**N**HAYNQKPNLEKNIKLTNIVE
hypothetical protein FKN15_057322	*Acipenser sinensis*	gb|MGH0157658.1|/1-303	MAENWKNCFE[x 227]EELICPICLHVFVEPIQLPCKHNFCRGCISEAWSKDTGMVRCPEC**N**HAYNQKPTLEKNLKLTNIVE
E3 ubiquitin-protein ligase TRIM8a	*Trichomycterus rosablanca*	ref|XP_062852088.1|/9-84	MAESWKNCFE-------EELICPICLHVFVEPVQLPCKHNFCRGCISEAWAKDTGLVRCPEC**N**HTYNQKPNLEKNIKLTNIVE
LOW QUALITY PROTEIN: E3 ubiquitin-protein ligase TRIM8b	*Brienomyrus brachyistius*	ref|XP_048843275.1|/1-76	MAENWKNCFE-------EELICPICLHVFSEPIQLPCKHNFCRGCISEAWAKDTSMVRCPEC**N**HAYNQKPSLEKNHKLSNIVE
TRIM8 ligase, partial	*Polyodon spathula*	gb|MBN3285331.1|/1-76	MAENWKNCFE-------EELICPICLHVFVEPIQLPCKHNFCRGCISEAWSKDTGMVRCPEC**N**HTYNQKPTLEKNLKLTNIVE
putative E3 ubiquitin-protein ligase TRIM8	*Arapaima gigas*	gb|KAL4612921.1|/1-76	MAENWKNCFE-------EELICPICLHVFSEPIQLPCKHNFCRGCISEAWSKDTGLVRCPEC**N**HAYNQKPSLEKNHKLSNIVE
hypothetical protein AGOR_G00114060	*Albula goreensis*	gb|KAI1894268.1|/1-76	MAENWKNWFE-------EELICPICLHVFVEPIQLPCKHNFCRGCISEAWSKDTAMVRCPEC**N**HAYNQKPTLEKNIKLTNIVE
E3 ubiquitin-protein ligase TRIM8a	*Danio rerio*	ref|NP_001265783.1|/1-76	MAESWRNCFE-------EELICPICLHVFVEPVQLPCKHNFCRGCISEAWAKDAANVRCPEC**N**HAYSQKPNLEKNIKLNNIVE
hypothetical protein AAFF_G00138100	*Aldrovandia affinis*	gb|KAJ8418101.1|/1-76	MADNWKNWFE-------EELICPICLHVFVEPIQLPCKHNFCRGCISEAWSKDTSMVRCPEC**N**HAYNQKPTLEKNIKLANIVE
unnamed protein product	*Gadus morhua 'NCC'*	emb|CAL8382151.1|/12-87	MAETWRNCFE-------EELICPICLHVFSDPIQLPCKHNFCRGCISEAWAKDSLLARCPEC**N**HAYSQKPSLEKNHKLSNIVE
putative E3 ubiquitin-protein ligase TRIM8, partial	*Clarias magur*	gb|KAF5890873.1|/1-76	MAEAWKNCFE-------EELICPICLHVFVDPVQLPCKHNFCHGCISEAWAKDVGILRCPEC**N**QPYKQKPSLDKNIKLINIVE
unnamed protein product	*Tetraodon nigroviridis*	emb|CAG14116.1|/6-81	MAETWRNCFE-------EELICPICLHVFSDPIQLPCKHNFCRGCISEAWAKDSSLARCPEC**N**HAYTQKPSLEKNHKLSNIVE
E3 ubiquitin-protein ligase TRIM8b	*Garra rufa*	ref|XP_073684017.1|/1-76	MAENWKNCLE-------EELICPICLNVFSEPVQLPCKHNFCRMCINEAWSKDTGMVRCPEC**N**HAYSQKPALEKNHKLSNIVE
Tripartite motif-containing protein 8	*Dicentrarchus labrax*	emb|CBN81612.1|/1-76	MAETWRNCFE-------EELICPICLHVFSDPIQLPCKHNFCRGCISEAWAKDSSLARCPEC**N**HAYTQKPSLEKNHKLSNIVE
E3 ubiquitin-protein ligase TRIM8b	*Pygocentrus nattereri*	ref|XP_017551569.1|/1-76	MADNWKNCLE-------EELICPICLHVFSEPVQLPCKHNFCRVCINEAWAKDGGMVRCPEC**N**HAYSQKPSLEKNHKLSNIVE
E3 ubiquitin-protein ligase TRIM8b	*Denticeps clupeoides*	ref|XP_028844081.1|/1-76	MAESWKNLFE-------EELICPICLHVFSEPIQLPCKHNFCRGCINEAWSKDAGVVRCPEC**N**HAYSQKPTLEKNHKLSNIVE
E3 ubiquitin-protein ligase trim8	*Goodea atripinnis*	gb|MEQ2175676.1|/11-86	MAETWRNCFE-------EELICPICLHVFSDPIQLPCKHNFCRGCISEAWAKDSSLARCPEC**N**HAYTQKPSLEKNHKLSNIVE
unnamed protein product	*Lampetra planeri*	emb|CAM9294253.1|/1-76	MAETWRNCFE-------EELICPICLHVFSDPIQLPCKHNFCRGCISEAWAKDSSLARCPEC**N**HAYTQKPSLEKNHKLSNIVE
unnamed protein product	*Knipowitschia caucasica*	emb|CAL9699232.1|/6-81	MAESWRNCFE-------EELICPICLHVFSEPIQLPCKHNFCRGCIGEAWAKDTTATRCPEC**N**HGYAQKPSLEKNHKLSNIVE
E3 ubiquitin-protein ligase TRIM8b	*Danio rerio*	ref|NP_001135847.1|/1-76	MAENWKNCLE-------EELICPICLNVFSEPVQLPCKHNFCRMCINEAWSKDAGMVRCPEC**N**NAYTQKPALEKNHKLSNIVE
hypothetical protein NHX12_025350	*Muraenolepis orangiensis*	gb|KAJ3608301.1|/12-87	MAETWRNCFE-------EELICPICLHVFSDPIQLPCKHNFCRGCISEAWAKDSSLARCPEC**N**HAYSQKPNLEKNHKLSNIVE
hypothetical protein DNTS_004354	*Danionella cerebrum*	gb|TRY74185.1|/8-83	MTESWRNCFE-------EELICPICLHVFVESVQLPCKHNFCRGCISEAWAKEAAGVRCPEC**N**HAYNQKPSLEKNIKLNNIVE
E3 ubiquitin-protein ligase TRIM8a	*Tachysurus vachellii*	ref|XP_060728167.1|/9-84	MAEAWKNCLE-------EELICPICLHVFVDPVQLPCKHNFCHGCISEAWAKDVGILRCPEC**N**QPYKQKPSLDKNIKLINIVE
E3 ubiquitin-protein ligase TRIM8b	*Trichomycterus rosablanca*	ref|XP_062859081.1|/1-76	MAENWKNCLE-------EELICPICLHVFSEPIQLPCKHNFCRVCINEAWAKDATMVRCPEC**N**HSYSQKPALEKNHKLSNIVE
E3 ubiquitin-protein ligase TRIM8b isoform X2	*Lampris incognitus*	ref|XP_056153321.1|/11-86	MAETWRNCFE-------EELICPICLHVFSDPIQLPCKHNFCRGCISEAWAKDSTLARCPEC**N**HAYTQKPSLEKNHKLSNIVE
E3 ubiquitin-protein ligase TRIM8b	*Clupea harengus*	ref|XP_012692597.1|/4-79	MAENWKNSFE-------EELICPICLHVFSEPIQLQCKHNFCRGCINEAWSKDTSVVRCPEC**N**HAYSQKPSLEKNHKLSNIVE
hypothetical protein F2P81_022522	*Scophthalmus maximus*	gb|KAF0025641.1|/6-81	MAETWRNCFE-------EELICPICLHVFSDPIQLPCKHNFCRGCISEAWAKDSSLARCPEC**N**HAYTQKPSLEKNHKLSNIVE
E3 ubiquitin-protein ligase TRIM8b	*Lates japonicus*	dbj|GAA6214476.1|/11-86	MAETWRNCFE-------EELICPICLHVFSDPIQLPCKHNFCRGCISEAWAKDSSLARCPEC**N**HAYTQKPSLEKNHKLSNIVE
putative E3 ubiquitin-protein ligase TRIM8	*Bagarius yarrelli*	gb|TSK14774.1|/1-76	MAETWKNCLE-------EELICPICLHVFSEPVQLPCKHNFCRVCINEAWAKEAAMVRCPEC**N**NAYTQKPALEKNHKLSNIVE
E3 ubiquitin-protein ligase TRIM8b	*Nelusetta ayraudi*	ref|XP_075889670.1|/11-86	MAETWRNCFE-------EELICPICLHVFSDPIQLPCKHNFCRGCISEAWAKDSTLARCPEC**N**HAYTQKPSLEKNHKLSNIVE
hypothetical protein CCH79_00003139, partial	*Gambusia affinis*	gb|PWA21766.1|/11-86	MAETWRNCFE-------EELICPICLHVFSDPIQLPCKHNFCRGCISEAWAKDSSLARCPEC**N**HAYTQKPSLEKNHKLSNIVE
hypothetical protein INR49_029467	*Caranx melampygus*	gb|KAG7227705.1|/11-86	MAETWRNCFE-------EELICPICLHVFSDPIQLPCKHNFCRGCISEAWAKDSSLARCPEC**N**HAYTQKPSLEKNHKLSNIVE
E3 ubiquitin-protein ligase TRIM8b	*Electrophorus electricus*	ref|XP_026866259.2|/1-76	MADNWKNCLE-------EELICPICLHVFSEPVQLPCKHNFCRVCINEAWAKDVSMVRCPEC**N**NAYSQKPTLEKNHKLSNIVE
E3 ubiquitin-protein ligase TRIM8b	*Osmerus eperlanus*	ref|XP_062330195.1|/10-85	SAETWRNCFE-------EELICPICLHVFSDPIQLPCKHNFCRGCISEAWAKDSTLARCPEC**N**HAYTQKPTLEKNHKLSNIVE
